# New Antimicrobials and New Therapy Strategies for Endocarditis: Weapons That Should Be Defended

**DOI:** 10.3390/jcm12247693

**Published:** 2023-12-14

**Authors:** Alessandra Oliva, Francesco Cogliati Dezza, Francesca Cancelli, Ambrogio Curtolo, Antonio Falletta, Lorenzo Volpicelli, Mario Venditti

**Affiliations:** Department of Public Health and Infectious Diseases, Sapienza University of Rome, 00185 Rome, Italy; francesco.cogliatidezza@uniroma1.it (F.C.D.); francesca.cancelli@uniroma1.it (F.C.); ambrogio.curt@uniroma1.it (A.C.); fallettaantonio0@gmail.com (A.F.); lorenzo.volpicelli@uniroma1.it (L.V.); mario.venditti@uniroma1.it (M.V.)

**Keywords:** infective endocarditis, ceftobiprole, ceftaroline, fosfomycin, long-acting lipoglycopeptides, dalbavancin, oritavancin, strategy, oral therapy

## Abstract

The overall low-quality evidence concerning the clinical benefits of different antibiotic regimens for the treatment of infective endocarditis (IE), which has made it difficult to strongly support or reject any regimen of antibiotic therapy, has led to a discrepancy between the available guidelines and clinical practice. In this complex scenario, very recently published guidelines have attempted to fill this gap. Indeed, in recent years several antimicrobials have entered the market, including ceftobiprole, ceftaroline, and the long-acting lipoglycopeptides dalbavancin and oritavancin. Despite being approved for different indications, real-world data on their use for the treatment of IE, alone or in combination, has accumulated over time. Furthermore, an old antibiotic, fosfomycin, has gained renewed interest for the treatment of complicated infections such as IE. In this narrative review, we focused on new antimicrobials and therapeutic strategies that we believe may provide important contributions to the advancement of Gram-positive IE treatment, providing a summary of the current in vitro, in vivo, and clinical evidence supporting their use in clinical practice.

## 1. Introduction

Infective endocarditis (IE) is a potentially lethal disease that always poses new diagnostic and therapeutic challenges. The yearly incidence is about 3–10 cases per 100,000 people, with an overall mortality of about 30% [1]. In 2019, the estimated incidence of IE was 13.8 cases per 100,000 subjects per year, and IE accounted for over 66,000 deaths worldwide [2]. The aetiological agents of IE can be Gram-positive or Gram-negative bacteria or, less frequently, fungi. Among them, Gram-positive staphylococci, streptococci, and enterococci represent 80–90% of all IE causes [3].

Notably, 2023 has been an incredible and singular year for scientific advancements in IE management, witnessing the proposal of new revised Duke criteria to help diagnose endocarditis [4] and the recent publication of the new official European guidelines for IE that update the old version published eight years ago [5,6].

Between the publication of the 2015 guidelines and the new ones, new antibiotic molecules such as ceftaroline, ceftobiprole, dalbavancin, and oritavancin were approved by the Food and Drug Administration (FDA) and the European Medicine Agency (EMA) to meet the needs of tailored therapy and, accordingly, new antibiotic strategies were investigated. Indeed, despite being approved for indications other than IE, real-world data on their use, alone or in combination, for the treatment of IE has accumulated over time, providing clinical evidence on their possible therapeutic benefits over traditional regimens [7,8,9,10,11].

Furthermore, these molecules are characterised by high bactericidal activity towards the majority of microorganisms that commonly cause IE and, most importantly, exhibit a high safety profile in comparison with glycopeptides, which still represent the recommended option for methicillin-resistant Staphylococci. Fosfomycin, an old antibiotic discovered in 1969, has gained renewed interest in this setting thanks to (i) its broad activity against both Gram-positive and Gram-negative pathogens, including resistant ones, (ii) its high anti-biofilm activity, and (iii) its ability to synergise with several antimicrobials.

After the publication of the 2015 guidelines, the only relevant published randomised clinical trial (BACSARM) on IE treatment explored the combination of daptomycin and fosfomycin for the treatment of *S. aureus* IE [10]; however, only a few IE cases were included (approximately 8–10% for each arm).

Given that the complexity of endocarditis renders it difficult to set up a randomised controlled clinical trial to investigate the efficacy and safety of new drugs and antibiotic strategies, the evidence from the literature comes almost exclusively from observational retrospective studies [12]. Thus, the collection of clinical evidence concerning the efficacy and tolerability of new therapeutic strategies is highly needed to address the incertitude in the most recent guidelines and in current clinical practice [5].

Furthermore, the evolution of antibiotic therapy is moving more and more towards treatment individualization and shortening. In this context, the possibility of step-down oral treatments or replacement with long-acting antibiotics represent the new therapeutic frontiers in selected and eligible patients [13,14].

To build this narrative review, we focused on new antimicrobials and therapeutic strategies that we believe may provide important contributions to the advancement of Gram-positive IE treatment, providing a summary of the current in vitro, in vivo, and clinical evidence supporting their use in the clinical practice. Some of these strategies are also recommended in the new guidelines, such as the use of a combination of daptomycin and fosfomycin or ceftaroline for the treatment of staphylococci- or enterococci-induced IE [5].

Since several other antimicrobials retain fundamental roles in the treatment of IE caused, for instance, by streptococci or susceptible *E. faecalis*, our review does not aim to substitute these consolidated and effective regimens with the new drugs. Rather, we attempted to summarise the potential therapeutic weapons we currently possess for the treatment of IE, such as ceftaroline, ceftobiprole, fosfomycin, dalbavancin, and oritavancin, and their most relevant therapeutic associations.

We consciously decided not to include daptomycin alone in the new therapeutic strategies. Indeed, it has earned a place as an “established treatment” for IE in recent years, a role confirmed in recently published guidelines.

## 2. Materials and Methods

We discussed the main topics of the narrative review in several meetings. In the first round of discussion, the following topics were identified to be addressed in this review: (i) new antimicrobials and new strategies for the management of IE caused by the most common Gram-positive pathogens, which included: ceftobiprole, ceftaroline, dalbavancin, oritavancin in monotherapy, ceftobiprole or ceftaroline in combination with daptomycin, and fosfomycin in combination with ß-lactams or daptomycin; (ii) the in vitro activity and synergism of the new antimicrobials recognised; (iii) animal studies; (iv) clinical evidence concerning the efficacy of the selected antimicrobials, alone or in combination, in the treatment of IE due to Gram-positive pathogens.

Afterwards, we retrieved scientific evidence supporting the proposals of the review by means of a PubMed-MEDLINE literature search up to July 2023. The following search strategy and key terms were adopted: “endocarditis” or “infective endocarditis” or “bacteraemia” or “bloodstream infection” or “synergism” or “in vitro activity” or “experimental model” AND the name of each single antimicrobial were searched. The antimicrobials searched were “ceftobiprole” or “ceftaroline” or “fosfomycin” or “dalbavancin” or “oritavancin”.

We selected all available categories of articles, including randomised controlled trials (RCTs), multicentre or single-centre prospective observational studies, multicentre or single-centre retrospective observational studies, case series, case reports, and in vivo/in vitro preclinical studies.

During the subsequent shared discussions, we reviewed the articles’ relevance based on the authors’ opinions and the quality of evidence, established according to a hierarchical scale of study designs. Guidelines, systematic reviews, and meta-analyses were also consulted to address our proposals.

We excluded abstracts or articles not written in English. We did not consider any timeline limitations, but we mainly focused our research on studies published in the last 10 years.

In the final round of discussion, the last version of the manuscript was approved by all authors.

**Figure 1:**
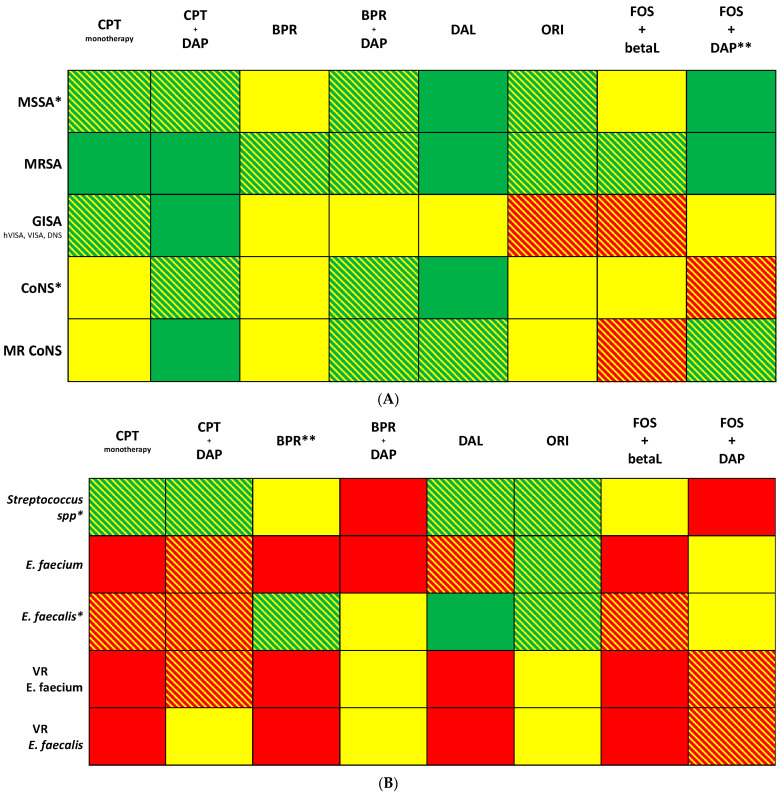
(**A**). Summary of the available in vitro, in vivo, and clinical evidence for a possible place in therapy for new antimicrobial strategies for *Staphylococcus* spp. infective endocarditis. *: Other regimens recommended for the treatment of *Staphylococcus* spp. IE due to strong and consolidated clinical evidence are not shown in this figure but are discussed in the text; **: clinical evidence derives from randomised clinical trials [10]. (**B**). Summary of available in vitro, in vivo, and clinical evidence for a possible place in therapy for new antimicrobial strategies for *Streptococcus* spp. and *Enterococcus* spp. infective endocarditis. *: Other regimens recommended for the treatment of *Streptococcus* and *E. faecalis* spp. IE due to strong and consolidated clinical evidence are not shown in this figure but are discussed in the text. ** As for *E. faecalis*, the suggested green/yellow colour refers only to clinical evidence for BPR in combination with ampicillin.

The review is structured as follows: Section 3 (Section 3.1, with corresponding Table 1; Section 3.2, with corresponding Table 2; Section 3.3, with corresponding Table 3; Section 3.4, with corresponding Table 4; Section 3.5, with corresponding Table 5); Section 4, with corresponding Table 6; Section 5, with corresponding Figure 1A,B; Section 6.

Legend of color. Green: evidence supported by in vitro, animal, and preliminary clinical studies; Green–yellow lines: evidence supported by in vitro activity, animal studies, and case report series; Yellow: evidence supported by in vitro activity and animal studies but lacking clinical evidence; Yellow–red lines: poor in vitro data, no in vivo data, no clinical data; Red: absence of in vitro, animal, and clinical data and/or no drug activity.

Abbreviation. MSSA: methicillin-susceptible *S. aureus*; MRSA: methicillin-resistant *S. aureus*; CoNS: coagulase-negative *Staphylococci*; VISA: vancomycin-intermediate *S. aureus*; hVISA: heterogeneus vancomycin-intermediate *S. aureus*; DNS: Damptomycin unsusceptible; VR: vancomycin-resistant. CPT: ceftaroline; DAP: daptomycin; BPR: ceftobiprole; DAL: dalbavancin; ORI: oritavancin; FOS: fosfomycin

## 3. New Antimicrobials

### 3.1. Ceftobiprole

#### 3.1.1. Mechanism of Action and Indication

Ceftobiprole (BPR) is a fifth-generation, novel broad-spectrum cephalosporin with a mechanism of action that involves binding to penicillin-binding proteins (PBPs), inhibiting cell growth and leading to bacterial cell death. A peculiarity of BPR is its ability to bind PBP2a, PBP2x, and PBP4, with increased activity against methicillin-resistant *Staphylococcus aureus* (MRSA), penicillin-resistant *Streptococcus pneumoniae* (PRP), and *Enterococcus faecalis*, respectively, as well as Gram-negative microorganisms, including non-extended spectrum β-lactamase (ESBL), non-AmpC and non–carbapenemase-producing *Enterobacterales*, and *Pseudomonas aeruginosa* [15,16,17,18,19,20].

Studies investigating BPR in vitro synergisms and experimental models of IE are discussed in Supplementary Material Sections S1.1 and S1.2 [18,21,22,23,24,25,26,27,28,29,30,31,32,33,34].

BPR is currently approved by the European Medicines Agency (EMA) for the treatment of community-acquired pneumonia (CAP), non-ventilator-associated hospital-acquired pneumonia (HAP), and acute bacterial skin and skin structure infections (ABSSSIs), including diabetic foot infections.

#### 3.1.2. Clinical Evidence in Infective Endocarditis

The evidence available in the literature concerning the use of BPR in IE consists of a double-blinded, randomised, controlled non-inferiority study and observational and retrospective studies, case series, and case reports [7,35,36,37,38,39,40] (Table 1).

**Table 1:** Clinical studies investigating the treatment of infective endocarditis with ceftobiprole.

Authors	Study Design	Endpoint	N° Patients/ IE Type	Pathogens	Dosage and Duration	Combination	Outcomes	Safety
Holland, T.L. et al., 2022 * [39]	Randomised double-blind trial (ERADICATE study) BPR vs. DAP ±Aztreonam	Clinical success Success required survival, symptom improvement, SAB clearance, no new SAB complications, and no use of other potentially effective antibiotics	390 SAB 192 BPR vs. 198 DAP IE 33 BPR: 20, 15 right-sided, 5 left-sided DAP: 13, 10 right-sided, 3 left-sided	MSSA 287 MRSA 94	500 mg/6 h up to 42 d	±Aztreonam	Overall clinical success: 69.8% in BPR vs. 68.7% for DAP There were no significant differences in mortality or microbiological eradication between treatment groups	≥1 AE: 63% BPR vs. 59% DAP
Gentile, I. et al., 2023 [7]	Multicentre observational and ambispective study Mono vs. combination therapy	Clinical success: As a composite of the clinical cure, improvement or de-scalation feasibility in 30 d FU	195, 34% mono vs. 66% combination (pneumonia 74%; BSI 19%; SSTI 5%; bone infection 4%) IE 7 (4%), all combination	Polymicrobial infection (25%) MSSA (11%) MRSA (38%) **In IE subgroup:** 2/7 MRSA; 5/7 MRCoNS	No data reported	MER 31% **In IE subgroup:** DAP 6/7 and LNZ 1/7	Overall, clinical success 79%, microbiological cure 87%, 8 infection recurrences **In IE subgroup:** Clinical success 29% Microbiological cure 29% (presumed eradication)	7 AE (2 rash, 2 myoclonus, 1 allergic reaction, 1 seizure, 1 CDI) 4 AE (rash or myoclonus) were BRP + DAP
Mahmoud, E. et al., 2020 [36]	Case series	N/A	6 BSI (2 osteomyelitis,1 IE, 1 CLABSI, 1 SSTI, 1 pneumonia) IE 1 NVE	MRSA	No data reported on the dosage 31 d	All VAN	All demonstrated microbiological and clinical cure at 14 d	No data reported
Tascini, C. et al., 2020 [37]	Case series BPR + DAP or BPR	N/A	IE 12 8 PVE, 3 NVE, 1 CIED-IE 5 surgeries for vegetation size (n.3) or severe valve disfunction with heart failure (n. 2) 9/12 previous therapy BPR + DAP 11 BPR 1	25% polymicrobial 33.3% MSSA 33.3% MRSA	No data reported on dosage Up to 84 d	91.7% DAP	Clinical success: 10/12 (83%) Microbiological cure: In 9/12 (75%) cases, patients were switched to BPR following failure of the previous antimicrobial regimen. In 3/3 patients in which BPR was administered because of persistently positive blood culture, bacteraemia clearance was rapidly achieved.	No data reported
Zhanel, G.G. et al., 2021 [38]	Case series Mono and combination therapy	N/A	38 infections 42.1% IE 23.7% BJIs 15.8% HABP 5.3% SSTI 2.6% CNS 2.6% DRI 2.6% BSI 9 mono and 29 combination	MRSA	500 mg/8 h No data on duration	Combination therapy 76.3%: - DAP 21/29 - VAN 7/29 - FLUORO 1/29	Overall, clinical success 84.8%, microbiological cure 97.0% **In IE subgroup:** - Microbiological cure: 14/16, 2/16 unknown - Clinical success: 11/16, 4/16 unknown; 1/16 death	2.6% AE (gastrointestinal symptoms)
Giuliano, S. et al., 2023 [40]	Case series	N/A	21 BSI 13 left-sided IE 8 PVE, 5 NVE, 1 PVE + NVE	*E. faecalis* AMP S	15/21 500 mg/8 h 3/21 500 mg/12 h 3/21 350 mg/8 h Among patients with IE, the mean duration of the ABPR regimen was 27.8 ± 14.5 days. In patients with *E. faecalis* bacteraemia, the mean duration of ABPR treatment was 20.4 ± 11.1 days.	All ampicillin	Overall clinical success 81%, microbiological cure 86% **In IE subgroup:** - Clinical success: 9 (6 PVE, 3 NVE) - Microbiological cure: 10 (5 PVE, 5 NVE) 1 relapse in NVE (pt did not adhere to the partial oral treatment)	9% experienced ABPR-related side effects (seizure and skin rash)
Oltolini, C. et al., 2016 [35]	Case report	N/A	1 PVE	MRSA	250 mg/2 h then 500 mg/8 h according to GRF 11 weeks	DAP	Clearance of bacteraemia Complete disappearance of the vegetation at echocardiography IE recurrence (it was not attributable to antibiotic failure but to EVS with the implantation of a new prosthesis during an uncontrolled infection status and also the recurrence of PVE and the need for chronic antibiotic therapy)	No data reported

Abbreviations: ABPR: ampicillin plus ceftobiprole combination; BJI: bone and joint infection; BPR: ceftobiprole; BSI: bloodstream infection; CIED-IE: cardiovascular implantable electronic device endocarditis; CDI: clostridioides difficile infection; CLABSI: central line-associated bloodstream infection; CNS: central nervous system; DAP: daptomycin; DRI: device-related infection; IE: infective endocarditis; EVS: early valve surgery; FLUORO: fluoroquinolone; HABP: hospital-associated bacterial pneumonia; LNZ: linezolid; MRSA: methicillin-resistant *S. aureus*; MR CoNS: methicillin-resistant coagulase-negative Staphylococci; MSSA: methicillin-sensible *S. aureus*; NVE: native valve infection; PVE: prosthetic valve infection; SAB: *S. aureus* bacteraemia; SSTI: skin and soft tissue infection; VAN: vancomycin; N/A: not applicable: AE: adverse events. Definitions: Clinical success was defined as clinical improvement with resolution of all signs and symptoms of infection during BPR treatment or at the end of therapy. Microbiological cure was defined as negative follow-up blood cultures after the index-positive blood culture at some point during treatment and a negative valve culture in patients who underwent surgery. Notes: * all the ERADICATE study results were published at the end of September 2023 and were not included in the review. As for the results published in 2022, the study confirmed the non-inferiority of BPR compared to DAP.

The recent ERADICATE study, a randomised double-blind trial, compared the efficacy of BPR versus daptomycin ± aztreonam in the treatment of *S. aureus* bacteraemia (SAB) (*n* = 390), including ABSSSI, osteomyelitis, and native-valve IE (8.5%). Daptomycin (DAP) was administered at a dosage ranging from 6 mg/Kg to 10 mg/Kg q24h, while BPR was given at a dosage of 500 mg q6h from Day 1 to Day 8 and 500 mg q8h from Day 9 onwards, with dose adjustments according to renal function. The study showed the non-inferiority of BPR compared to DAP in terms of mortality rates, microbiological eradication, and the occurrence of new complications associated with bacteraemia (overall clinical success: 69.8% in BPR-regimen vs 68.7% in DAP-regimen) [39,41].

In a recent Italian multicentre observational study on the real-life use of BPR, seven cases of IE were described: two from MRSA and five from methicillin-resistant coagulase-negative staphylococci (MR-CoNS). BPR was always used in combination with DAP (*n* = 6) and linezolid (*n* = 1). In this study, only two out of seven patients with IE achieved clinical success, with a mortality rate of 28.6%, while overall microbiological and clinical success was obtained in 29% of patients [7].

Tascini et al. described the use of BPR in 12 patients with EI caused by Staphylococcus spp., including MRSA (*n* = 4). Three patients had polymicrobial IE. The majority of patients (83%) were switched to BPR due to the failure of previous antimicrobial regimens, mostly represented by DAP. BPR was administered in combination with DAP in 11/12 patients, while in one patient, BPR was administered as monotherapy. The cure rate was 83% (10/12 patients). Notably, the addition of BPR resulted in a rapid microbial clearance in all the three patients with persistently positive blood cultures under previous treatments [37].

Taking into account BPR’s pharmacokinetic–pharmacodynamic (PK–PD) profile, its microbial activity against *E. faecalis* by means of a high level of enterococcal PBP saturation, its synergism in combination with amoxicillin, and its enhanced activity against biofilms, Giuliano et al. investigated the use of BPR in combination with ampicillin (AMP) in a case series of 21 patients hospitalised for infections due to *E. faecalis*, including IE (*n* = 13). Clinical success was reached in 81% patients, with a microbiological cure obtained in 86% of patients. In the EI subgroup, clinical and microbiological success was reached in 69% and 77% of patients, respectively [40]. Experiences from case reports and case series in the literature also suggest the effectiveness of BPR as a monotherapy or as a combination regimen with DAP in achieving the microbiological eradication of MRSA EI [35,36,38].

Overall, we recorded 70 IE episodes caused mostly by *Staphylococcus aureus* (both methicillin-resistant and susceptible (MSSA)) and 13 cases of left-side IE due to AMP-S *E. faecalis*. The cases occurred in both native and prosthetic valves. Notably, the RCT ERADICATE included mostly right-sided IE. The outcomes were frequently favourable, with a good percentage of cases ending in microbiological and clinical cure.

### 3.2. Ceftaroline

#### 3.2.1. Mechanism of Action and Indication

Ceftaroline (CPT) is an intravenous fifth-generation cephalosporin which inhibits the bacterial cell wall by irreversibly binding PBPs. As in the case of ceftobiprole, its molecular structure confers an increased binding affinity to PBP-2a, improving its activity against MRSA [42]. CPT also exhibits in vitro activity against CoNS, streptococci (including *S. pneumoniae* and *S. pyogenes*), *Moraxella catarralis*, *Haemophilus influentiae*, and Gram-negative bacteria including *Klebsiella* spp. and *Escherichia coli*. Notably, the in vitro activity includes vancomycin-intermediate *S. aureus* (VISA) and cephalosporine-resistant *S. pneumoniae* [43]. In contrast, CPT seems to have no activity against *E. faecium* and a variable activity against *E. faecalis* [44].

The data available in the literature investigating CPT in vitro synergisms and experimental models of IE are discussed in Supplementary Material, Sections S2.1 and S2.2 [45,46,47,48,49,50,51,52,53,54,55,56,57,58,59,60,61,62].

CPT is currently approved by the FDA and EMA for the treatment of ABSSSI and CAP caused by susceptible microorganisms including MRSA. It is also approved in case of ABSSSI and CAP with intercurrent bacteriemia due to susceptible microorganisms with caution in MRSA bacteriemia in course of CAP [63].

#### 3.2.2. Clinical Evidence in Infective Endocarditis

Several studies investigating the treatment of bacteriemia due to MRSA consider CPT an option even in IE populations. However, the results in IE were often not reported or were discussed separately, although two multicentre observational retrospective studies and one case series reported results only for IE. Relevant clinical studies and case reports on the use of CPT in IE are summarised in Table 2.

Only one RCT enrolling patients with MRSA bloodstream infection (BSI) (*n* = 40) included IE (*n* = 7) and randomised patients in combination therapy with CPT + DAP (600 mg/8 h or adjusted for renal function) or DAP/VAN monotherapy. The IE patients were randomised as follows: three were in the combination group vs. four in the monotherapy group (3 VAN and 1 DAP). Overall, the study showed that combination therapy was associated with a significantly lower in-hospital mortality rate (0% vs. 26%; *p* = 0.029), which was also reflected in the IE subgroup; the excess mortality observed in the monotherapy arm during the interim analysis led the investigators to stop the study early [8]. The study was a pilot clinical trial which did not reach an appropriate sample size; consequently, the results did not provide any strong evidence and no definitive conclusions could be drawn.

Brandariz-Nunez and colleagues described 70 IE cases caused by different pathogens (MSSA, MRSA, MS and MR CoNS, AMP-S *E. faecalis*, *Streptococcus* spp.), all of which were CPT in vitro susceptible, with a 30% overall in-hospital mortality rate and a 38.6% treatment failure ate at 42 days. CPT was used in combination, mostly with DAP, at a dosage of 600 mg every 8 h or 12 h (or adjusted based on renal function) [64].

The CAPTURE study, a multicentre observational retrospective cohort, reported 55 IE cases due to different Gram-positive bacteria, mostly MRSA (80%), with an overall clinical success of more than 70% and a high success rate when CPT was administered as a first, second, or later line therapy. CPT was used in 32 patients as a combination therapy, mostly with DAP or vancomycin (VAN) [65].

Three multicentre retrospective studies including patients with various Staphylococcal infections and treated with CPT both in combination or monotherapy reported data on IE patients’ outcomes: clinical success was observed in 69.7% and 78% of cases in two studies [56,66], with mortality rates of 22.9%, 7%, and 11%, respectively [56,66,67].

Zasowski and colleagues observed in both MRSA BSI and IE populations that CPT monotherapy was not inferior to DAP in terms of composite failure, expressed in terms of 30 d mortality, persistent bacteraemia > 7 d, and 60 d BSI recurrence [68].

In a large multicentre retrospective study, there was no significant difference in terms of the mortality rate, hospital readmission, or BSI recurrence between combination therapy with DAP plus CPT (with no data reported on dosage) and the standard of care monotherapy (mostly VAN) in the treatment of 171 patients with MRSA BSI, of which 70 had IE [69].

Few single-centre observational studies reported positive clinical and/or microbiological outcomes in MRSA BSI populations, with or without specific data on the IE subgroups [70,71,72,73,74,75,76]. Additionally, several case series and complicated case reports showed microbiological cure and clinical success in IE patients treated with CPT as a monotherapy or in combination [56,72,77,78,79,80,81,82,83,84,85,86,87,88,89,90,91,92].

While the majority of studies described the use of CPT in combination, mostly with DAP but also with VAN, some studies investigated CPT use in monotherapy versus combination therapy. In 2017, Zasowski [93] and colleagues showed no statistical differences in mortality, microbiological cure, and clinical success between CPT monotherapy [most common dose 600 mg (61.8%) and frequency every 8 h (58.4%)] and combination therapy in 126 patients with MRSA BSI included in the efficacy population group, with 31 cases of IE. Likewise, a recent study observed no statistically significant differences in the composite outcomes of inpatient infection-related mortality, 60 day readmission, and 60 day BSI recurrence in MRSA BSI patients treated only with combination therapy (DAP + CPT) versus de-escalation to monotherapy (DAP/CPT/VAN) after a start with DAP + CPT [94].

Overall, the safety profile of CPT seemed to be similar to that of other beta-lactams also used in prolonged treatment for IE. In a recent systematic review, authors found 9% (83 out of 933) of adverse events were related to the use of CPT, mostly gastrointestinal events, rashes, and neutropenia [95]. In our review, we also found several cases of C. difficile infections, eosinophilia, and thrombocytopenia and a few cases requiring CPT withdrawal (Table 2).

Overall, we recorded 677 IE cases caused mostly by MRSA and involving both native and prosthetic valves (right and left sides) as well as CIEDs. The outcomes, when reported, were frequently positive, with microbiological and clinical cure.

**Table 2:** Clinical studies investigating the treatment of infective endocarditis with ceftaroline.

Authors	Study Design			Endpoint		N° Patients/ IE Type	Pathogens	Dosage and Duration	Combination	Outcomes	Safety
Geriak, M. et al., 2019 [8]	Randomised clinical trial DAP + CPT vs. VAN/DAP	Primary endpoints: duration of bacteraemia and in-hospital mortality Secondary endpoints: 60 d and 90 d mortality, hospital stay	40 BSI, 17 DAP + CPT vs. 23 VAN/DAP (VAN 21, DAP 2) 7 IE, 3 DAP + CPT vs. 4 VAN/DAP (1 bilateral, 1 right-sided, 1 aortic PVE, 1 mitral NVE, 1 aortic NVE, 2 CIED)	MRSA	CPT 600 mg 8 h (or adjusted for GFR) Mean 11 d	DAP 8 mg/kg/24 h	Overall, 30 d, 90 d, and in-hospital mortality: DAP + CPT 0 vs. VAN/DAP 6, 0 vs. 7, 0 vs. 6 Treatment failure *: 1 vs. 3 **IE subgroup:** in-hospital mortality, 0 vs. 2	No AE reported			
Casapao, A.M. et al., 2014 [66]	Multicentre observational retrospective study CPT in various infections			Clinical and microbiological success/failure, hospital length of stay, AEs, 30 d readmission, in-hospital mortality, and 30 d mortality.		527 infections 148 (28.1%) BSI 35 IE	138 SAB with 92% MRSA in IE group 6 hVISA	Overall, 85.6% 600 mg/12 h, 14.4% 600 mg/8 h Median 9 (4–15) in BSI group	29.2% combination therapy, 42% of which was with metronidazole	**In IE subgroup:** Clinical failure 30.3% Mortality 22.9%	In the BSI group: 12.8% AE
Arshad, S. et al., 2017 [76]	Retrospective case-control study CPT vs. VAN vs. DAP			Composite failure: 30 d mortality from infection onset, 42 d BSI recurrence, or 30 d readmission after the end of treatment		132 BSI, monotherapy 30 CPT vs. 46 VAN vs. 56 DAP 39 IE 7 vs. 13 vs. 19	MRSA	No data reported	No data reported	Overall, 30 d mortality: CPT group 13% vs. DAP group 24% and VAN group 11% (*p* = 0.188) Overall and in the **IE subgroup**, no statistically significant difference in 30 d mortality, 42 d recurrence, and 30 d readmission	No data reported
Britt, R.S. et al., 2017 [67]	Multicentre observational retrospective study CPT in various infections			AEs within 30 d of therapy initiation All-cause in-hospital mortality		764 infections 46 IE	No data reported	No data reported	No data reported	Overall, in hospital mortality 5%, 30 d readmission 33% **IE subgroup** mortality 11%, 30 d readmission 28%	AE < 1% (eosinophilia, leukopenia, fibromyalgia, myalgia and myositis, and polymyalgia)
Zasowski, E.J. et al., 2017 [93]	Multicentre observational-retrospective study CPT mono vs. combination therapy in BSI			Safety and efficacy outcomes		211 BSI, 126 included in the efficacy population 31 IE 20 CPT mono vs. 11 combination therapy	MRSA 1% VAN resistant strain	In efficacy population, most common dose 600 mg (60.3%) and frequency every 8 h (52.4%) In efficacy population, median 13 d (IQR 5–21)	DAP combination in 75.7%	In efficacy population no statistical differences between monotherapy and combination. Clinical success ^§^ 86/126 (68.3%) monotherapy 69.7% vs. combination 64.9%, BSI clearance 115/126 ^§§^ (91.3), 88.8% vs. 97.3%, Mortality 28/126 (22.2%), 19.1% vs. 29.7%	Overall, 16 AE (6 CDI, 7 rash, 3 neutropenia)
Cortes-Penfield, N. et al., 2018 [71]	Observational retrospective study DAP + CPT vs. DAP in BSI			Duration of bacteraemia, mortality, BSI recurrence		17 BSI, 5 IE 12 DAP + CPT and 5 DAP	MRSA	No data on dosage Mean 32.5 d	DAP median dose 7.6 mg/kg/24 h (5.7–13.8)	Overall, shorter duration of bacteraemia in DAP + CPT group **IE subgroup** mortality 3/5	No data reported
Destache, C.J. et al., 2019 [65]	Multicentre observational retrospective study CPT mono or combination therapy in IE			Clinical outcomes		55 IE, 26 right-sided, 25 left sided, 4 bilateral	MRSA 44/55 MSSA 4 CoNS 4 *E. faecalis* 1 *Streptococcus* 1	Mainly 600 mg/12 h Mean (SD) 13.4 d (9.7)	32, most common drugs (>5% of pt) DAP (n. 19), VAN (n. 9), RIF (n. 7). Other drugs: CFZ, LVX, LNZ, GEN, AMP.	Overall, clinical successes 39 (70.9%): monotherapy 19/23 (82.6%), combination 20/32 (62.5%) High success rate with CPT as first or second line therapy	2 AE (AKI and rash) with CPT withdrawal
McCreary, E.K. et al., 2019 [69]	Multicentre observational retrospective study DAP + CPT vs. SoC (case-control)			All-cause mortality, duration of bacteraemia, and BSI recurrence		171 BSI, 58 DAP + CPT vs. 113 SoC (VAN or DAP), 70 EI, 23 vs. 47	MRSA	No data reported	No data reported	No statistically significant difference in all-cause 30 d mortality and 90 d BSI recurrence	No data reported
Ahmad, O. et al., 2020 [70]	Retrospective case-control study VAN or DAP vs. VAN/DAP +CPT			Treatment outcomes: in-hospital mortality, BSI recurrence, 30 d readmission, AKI, leukopenia		30 BSI, 15 VAN/DAP vs. 15 VAN/DAP + CPT 21 IE, all NVE (14 vs. 7)	MRSA	600 mg/8–12 h Median 6 weeks	VAN 15–20 mg/kg/8–12 h DAP 8–10 mg/kg/24 h	No difference in AKI, leukopenia, BSI recurrence, 30 d readmission, or mortality	No AE reported
Morrisette, T. et al., 2020 [75]	Observational retrospective study DAP vs. DAP + CPT			Composite success: 30 d mortality, 60 d recurrence, worsening of respiratory status, change in therapy due to failure		29 BSI with septic pulmonary emboli, 14 DAP vs. 15 DAP + CPT 24 IE, all NVE (11 vs. 13)	MRSA	600 mg/8 h Median 11 d (9–12)	DAP median 9.9 mg/kg (8.8–9.8) duration median 36 d (22–42)	No difference in the primary outcome of compositive success	1 AE (thrombocytopenia) with CPT withdrawal
Johnson, T.M. et al., 2021 [73]	Observational retrospective study DAP + CPT vs. SoC			Clinical failure: MRSA-related mortality and 60 d recurrent infection		60 BSI, 30 DAP + CPT vs. 30 SoC, 22 IE, 15 vs. 7 (14 left-sided, 6 right-sided, 2 bilateral)	MRSA	1800 mg/24 h (or adjusted for GFR) DAP + CPT median 7 d (3–11)	DAP 10 mg/kg/24 h	Overall, clinical failure DAP + CPT 20% vs. SoC 43%, 60 d BSI recurrence 0% vs. 30%, 90 d mortality 27% vs. 23%, DAP + CPT inversely associated with clinical failure 90 d (*p* = 0.03)	No statistically significant AE reported
Nichols, C.N. et al., 2021 [94]	Observational retrospective study DAP + CPT vs. de-escalation with DAP/CPT/or VAN			Composite endpoint: inpatient infection-related mortality, 60 d readmission, and 60 d BSI recurrence		140 BSI, 66 DAP + CPT vs. 74 de-escalation in monotherapy DAP/CPT/VAN 63 IE, 37 vs. 26	MRSA	No data on dosage Median 56 d in combination group	DAP	No differences between combo and monotherapy for inpatient infection-related mortality, 60 d readmission, or 60 d BSI recurrence	In the combination group, 2 AE (bone marrow suppression, oedema)
Zasowski, E.J. et al., 2022 [68]	Multicentre observational retrospective study CPT vs. DAP monotherapy			Composite treatment failure: 30 d mortality, BSI duration ≥ 7 d on study drug, and 60 d MRSA BSI recurrence.		270 BSI, 83 CPT and 187 DAP 82 IE 27 vs. 55	MRSA	Most common dose 600 mg (68.7%) and frequency every 12 h (56.6%) Median 10 d (IQR 5–18)	No Monotherapy DAP median 8.5 mg/kg 24 h	In all populations and the **IE subgroup**, CPT not inferior to DAP No differences in any endpoints	Overall, 17 AE (9 rash, 4 CDI, 5 others) No data on CPT discontinuation was reported
Brandariz-Nunez, D. et al., 2022 [64]	Observational retrospective study CPT combination in IE			Treatment failure: presence of fever or positive BC at 7 d, positive BC recurrence, early antibiotic withdrawal due to lack of clinical response, AE or death		70 IE, 30 NVE, 36 PVE, 10 ICED-IE	MRSA 6/26; MR CoNS 15/26; *E. faecalis* AMP-S 5; *Streptococcus* 5	600 mg/8–12 h (or adjusted on GFR) Mean 21.26 d (DS 16.17)	70/70 combination DAP (n.52), GEN (n.18), RIF (n.6)	Overall, 42 d in-hospital mortality 30%; 42 d treatment failure 38.6%	6 AE with 4 CPT discontinuation
Kufel, W.D. et al., 2023 [74]	Observational retrospective study CPT + VAN in BSI			Effectiveness and safety Bacteraemia clearance post-CPT initiation		30 BSI, 20 IE, 7 tricuspid, 7 mitral, 4 aortic and 2 multiple valves	MRSA	600 mg/8 h Median 16 d (IQR 13.2)	All combination, VAN median 1250 mg/24 h	Overall, microbiological cure 96.7%; 90 d readmission for MRSA BSI 6.7%, all-cause 90 d mortality 26.7%, MRSAB-related mortality^+^ 13.3%	2 AE (rash) with CPT discontinuation
Lin, J.C. et al., 2013 [92]	Case series			N/A		10 infections 5 IE, 4 probable and 1 possible. 1 right-sided, 1 CIED, 1 NV+ CIED-IE, 2 no vegetation	MRSA	600 mg/8 h (or adjusted por GFR) Between 3 d to 7 weeks	No data reported	**IE subgroup** Clinical cure 3/5 Microbiological cure 4/5	2 AE, 1 CDI, 1 fever + rash + eosinophilia with CPT discontinuation
Ho, T.T. et al., 2012 [91]	Case series CPT monotherapy			N/A		6 BSI, 3 IE Cases 1 and 2: middle-aged men with mitral NVE Case 3: middle-age woman with mitral NVE	MRSA	600 mg/8 h Case 1: 42 d Case 2–3: 3 weeks	No	**IE subgroup** Case 1–3: microbiological cure and clinical cure	No data reported
Polenakovik, H.M. and Pleiman, C.M. 2013 [78]	Case series			N/A		31 BSI, 10 IE, 3 left-sided, 6 right-sided, and 1 CIED-IE	MRSA	CPT 1200–1800 mg/24 h (1 case GFR dose-adjusted) Overall median 30.4 d (IQR 7–60)	4 IE combinations with DAP, RIF, GEN, LNZ	Overall, microbiological cure 64.5% (**IE** 9 pt); Clinical success 74.2% (**IE** 9 pt); Treatment failure ° 25.8% (**IE** 1 pt) Recurrence 9.7% (**IE** 1 pt); Death 6.5%	Overall, 2 AE (eosinophilia) without CPT discontinuation (1 IE) 3 AE (eosinophilic pneumoniae, rash, diarrhoea) with CPT discontinuation
Fabre, V. et al., 2014 [72]	Case series			N/A		29 BSI 18 IE 4 right-sided, 11 left-sided, 1 CIED, 2 LVAD	MRSA	600 mg/8 h (or adjusted on GFR) No data on duration	24 combination therapies: 22 with TMP-SMZ 10–15 mg/kg/24 h 2 with DAP	Overall, microbiological success: 26/29 (90%); Treatment success ^#^ with 6 months FU: 9 (31%); Treatment failure ^##^: 4 (13%) (1 death, 3 recurrence)	1 AE (rash) with CPT discontinuation
Tattevin, P. et al., 2014 [79]	Multicentre case series CPT in IE			N/A		8 IE 3 aortic PVE, 1 aortic PV plus pulmonary valve, 1 CIED, 1 mitral and aortic NVE, 1 aortic NVE, 1 CIED plus aortic NVE	5 MRSA 3 MR CoNS	From 400 mg/12 h to 800 mg/8 h Median 13 d (5–42)	3 combination DAP (n 2) RIF (n 1)	Clinical success: 5/8 Clinical failure: 3/8	No AE reported
Gritsenko, D. et al., 2017 [90]	Case series CPT + VAN			N/A		5 BSI, 2 IE, Case 2: 42 y man with tricuspid NVE Case 5: 50 y mitral NVE	MRSA	Case 2: 400 mg/12 h (adjusted for GFR) 6 weeks Case 5: 600 mg/12 h (then adjusted for GFR) 7 d	Case 2 and 5: combo with VAN Case 5: 7 d	**IE subgroup** Case 2: microbiological cure and clinical success Case 5: death	No data reported
Hornak, J.P. et al., 2019 [77]	Case series CPT + DAP in BSI			N/A		10 BSI 6 IE, 1 mitral NVE, 3 aortic NVE, 1 CIED, 1 LVAD	MRSA	4600 mg/12 h, 1600 mg/8 h, 1 400 mg/h 8. Overall, median time 9 d (IQR 6–24)	All IE combination with DAP	**IE subgroup** microbiological cure 6/6; no recurrence; 30 d mortality and in-hospital mortality 1/6	3 AE (rash, eosinophilia, thrombocytopenia) without CPT discontinuation 1 eosinophilia in IE group
Rose, W.E. el al., 2012 [89]	Case report Failure with DAP			N/A		1 right atrial vegetation	MRSA and DNS	200 mg/12 h (haemodialysis dose-adjusted) 54 d	DAP 10 mg/kg/24 h	Microbiological cure and clinical success after failure with 11 d of monotherapy with DAP 6 mg/kg 48 h	No data reported
Jongsma, K. et al., 2013 [88]	Case report			N/A		1 tricuspid and aortic NVE	MRSA and DNS	600 mg/12 h 44 d	No	No resolution after 23 d of DAP and VAN, debridement on 19 d, microbiological cure at 7 d after CPT start, clinical success	No data reported
Sakoulas, G. et al., 2013 [87]	Case report Failure with AMP-based regimens			N/A		1 aortic NVE	HLGR *E. faecalis*	600 mg/8 h 6 weeks	DAP 8 mg/kg/24 h	Microbiological cure and clinical success achieved after failure with CRO + AMP (6 weeks) and then DAP + AMP (7 d). 2 weeks after CPT + DAP start, aortic valve replacement was performed	No data reported
Baxi, S.M. et al., 2015 [86]	Case-report CPT + DAP			N/A		1 mitral NVE	MRSA VISA and DNS	400 mg/12 h 6 weeks of CPT + DAP	DAP 10 mg/kg after dialysis	Negative BC from day 11 of DAP + CPT, remain negative at 28 d after discontinuation	No AE reported
Cunha, B.A. et al., 2015 [85]	Case report Persistent bacteraemia with DAP			N/A		1 aortic PVE	MRSA	600 mg/12 h 6 weeks	DAP 10–12 mg/kg/24 h	Persistent bacteraemia for 14 d under DAP 10 mg/kg 24 h BC negative after 4 d of DAP+ CPT, no recurrence	No data reported
Sundaragiri, P.R. et al., 2015 [84]	Case report			N/A		1 tricuspid NVE	MRSA	No data reported	No data reported	9 d valve replacement Death	No data reported
Duss, F.R. et al., 2019 [83]	Case report Persistent bacteraemia with VAN			N/A		1 left NVE	MRSA (MIC: VAN 1.5 mg/L, DAP 2 mg/L)	600 mg/12 h 6 weeks	DAP 10 mg/kg/24 h	BC positive under VAN 5 d; switch DAP + FOS; day 10 surgery and culture valve negative. After surgery CPT + DAP for 6 weeks. Negative BC and persistent negative at 6 months FU	No data reported
Jilani, T.N. and Masood, S.O. 2018 [82]	Case report Failure with DAP and VAN			N/A		1 pulmonic NVE	MRSA	600 mg/8 h 4 weeks after 2 weeks of VAN and DAP	No	Microbiological cure after 2 d of CPT and clinical success	No data reported
Lin, S.Y. et al., 2021 [81]	Case report Failure with DAP and VAN			N/A		1 mitral NVE	hVISA	600 mg/12 h 5 weeks	DAP 9 mg/kg/24 h	Microbiological cure and clinical success achieved after failure with monotherapy VAN (14 d) and then DAP (7 d)	No data reported
Warren, E.F. et al., 2022 [80]	Case report CPT+ nafcillin			N/A		Case 1, tricuspid NVE Case 2, CIED-IE	MSSA	Case 1 600 mg/8 h Case 2 600 mg/12 h (GFR dose-adjusted) Case 1: 11 d Case 2: 7 d	Case 1 and 2: nafcillin 12 g 24 h	Microbiological cure and clinical success	No data reported

Abbreviations: CPT: ceftaroline; AE: adverse event; MRSA: methicillin-resistant *Staphylococcus aureus*; IE: infectious endocarditis; hVISA: heterogeneus vancomycin-intermediate *S. aureus*; BSI: bloodstream infection; SAB: *S. aureus* bacteraemia; d: day; VAN: vancomycin; DAP: daptomycin; CDI: C. difficile infection; AKI: acute kidney injury; CoNS: coagulase-negative staphylococci; MSSA: methicillin-susceptible *S. aureus*; LNZ: linezolid; LVX: levofloxacin; CFZ: cefazolin; GEN: gentamicin; AMP: ampicillin; RIF: rifampicin; PVE: prosthetic valve endocarditis; NVE: native valve endocarditis; CIED-IE: cardiovascular implantable electronic device endocarditis; SoC: standard of care; GFR: glomerular filtration rate; BC: blood culture; MR CoNS: methicillin-resistant coagulase-negative staphylococci; MRSAB: methicillin-resistant *S. aureus* bacteraemia; N/A: not applicable; FU: follow up; VISA: vancomycin-intermediate *S. aureus*; MRSE: methicillin-resistant *Staphylococcus epidermidis*. Definitions: Clinical success/cure was defined as clinical improvement with resolution of all signs and symptoms of infection during CPT treatment or at the end of therapy, unless otherwise specified. Casapao AM et al. and Destache CJ et al. defined clinical success as above or as a clinical improvement with no further need for escalation while on CPT treatment or during hospitalization [65,66]. Clinical failure was defined as inadequate response or resistance to CPT therapy, worsening of the clinical conditions during the treatment, or new recurrent signs and symptoms at the end of CPT therapy [66]. Microbiological success/cure was defined as a documented negative blood culture result or BC clearance. Duration of bacteraemia was calculated as the number of days between the first positive blood culture and the first negative blood culture without subsequent positive cultures. Bacteraemia recurrence was defined as at least one positive blood culture for MRSA after an initial microbiological cure. Notes: ^§^ Clinical success was defined as BSI clearance and cessation of BSI signs and symptoms (i.e., fever and leukocytosis) by the end of therapy or discharge and living patients at hospital discharge; ^§§^ Clearance of bloodstream infection was defined as a series of two consecutive negative blood cultures. * Patients with persistent bacteraemia for ≥5 days or deemed to be failing clinically on the regimen selected by the randomization process. +MRSAB-related mortality was defined as death prior to blood culture clearance or within 2 weeks following blood culture clearance using the date of the first positive blood culture as Day 1. ° Treatment failure was defined as any of the following: (i) persistent signs and symptoms of infection at the end of CPT therapy; (ii) persistent MRSAB defined as >7 days; (iii) recurrent MRSAB after the end of CPT therapy; (iv) death that could be attributed to ongoing infection (defined as MRSA-positive blood cultures at the time of death, death occurring before resolution of the signs and symptoms of MRSAB, or autopsy finding indicating MRSA infection as a cause of death); and (v) adverse drug reaction requiring cessation of CPT treatment. ^#^ Treatment success was defined as the absence of microbiologic or clinical recurrence at least 6 weeks after the end of therapy; ^##^ treatment failure was defined as recurrence of MRSA infection after completion of CPT therapy or death related to MRSA infection.

### 3.3. Dalbavancin

#### 3.3.1. Mechanism of Action and Indication

Dalbavancin (DAL) is a semisynthetic lipoglycopeptide derived from teicoplanin which is characterised by a unique PK profile with a prolonged half-life, lasting just over two weeks [96]. Similar to glycopeptides, DAL binds the C-terminal D-alanyl-D-alanine motif of peptidoglycan, inhibiting wall biosynthesis [97]. DAL exhibits excellent in vitro activity against the main Gram-positive pathogens, including vancomycin-susceptible enterococci, VanB *E. faecalis*, and VanB *E. faecium*, although it is inactive against VanA-phenotype enterococci [98]. This second-generation lipoglycopeptide exhibits potential penetration of and activity against the established biofilm produced by Gram-positive bacteria [99].

Studies investigating DAL in vitro synergisms and experimental models of IE are shown in Supplementary Material, Sections S3.1 and S3.2 [100,101,102,103].

Currently, DAL is approved for ABSSSI in adults by the FDA and the EMA. Recently, the approval was extended to pediatric ABSSSI [104,105]. In fact, the off-label application of this antibiotic in more deep-seated infections commonly caused by Gram-positive bacteria and requiring prolonged antimicrobial treatment is supported by an ever-growing body of evidence, and it can be used in conditions including osteomyelitis, prosthetic joint infections, endovascular device infections, BSI, and IE [96].

#### 3.3.2. Clinical Evidence in Infective Endocarditis

The available evidence in the literature concerning the application of DAL in IE is still mainly represented by observational and retrospective studies, case series, and case reports. No prospective randomised trial is available yet. Moreover, many data are only available in aggregate form because IE cases were a subgroup of larger studied populations. DAL prescription has been reserved primarily for the consolidation or completion phase of treatment in patients with already cleared bacteraemia. Published relevant clinical studies and cases on the use of DAL in IE are summarised in Table 3.

In a two-year retrospective cohort study, 27 patients with Gram-positive IE received primary or sequential DAL. The majority (88.9%) were previously treated with another with another antimicrobial and gaining bacteremia clearance antimicrobial agent for bacteraemia clearance. DAL was administered as a twice-weekly regimen [1500 mg loading dose (LD), then 1000 mg] in 63.0% of cases, with a median duration of 6 weeks. Failure was described in one patient with incomplete surgical control of cardiac device-related MRSA IE who received 30 weekly DAL infusions. Importantly, all cases received at least one DAL dose in hospital, but 23 continued DAL as OPAT [14].

The Italian multicentric study DALBITA retrospectively enrolled 206 patients treated with DAL, of which six had IE. In the whole cohort, MRSA (32%), CoNS (29%), and methicillin-susceptible *S. aureus* (MSSA) (18%) were the most frequent isolates, and 77.8% of patients received prior therapy for a median of 15 days. Clinical success was recorded in 83.3% of the IE subgroup [106].

In a system-wide retrospective analysis of 56 people receiving long-acting lipoglycopeptides, five had IE. Forty received DAL, fourteen received oritavancin, and two received both, but the outcomes of the two agents were not distinguishable. The success rate was 100% among the three IE cases included in the success/failure analysis [107].

A national cohort included 19 IE cases (nine native valve and ten prosthetic) among 75 patients. In the whole cohort, the main isolates were *S. aureus* (51.4%) and CoNS (44.4%); prior therapy was received in 98.7% of cases. DAL dosing for IE was a 1500 mg single or double dose, with a cure rate of 72.2%. Here, DAL was largely used as a rescue treatment, justifying the high failure rate [108].

In a retrospective multicentre study on real-life DAL use, 25 out of 101 subjects had IE. All received other antimicrobials before DAL and 64% received concomitant antibiotics while on DAL. The success rate was 92% among IE patients [109].

DALBACEN is a multicentre retrospective Spanish cohort that included 124 elderly, predominantly male patients with major comorbidities who received DAL for IE (46.8% native valve, 43.6% prosthetic valve, and 9.6% pacemaker lead IE). CoNS (38.7%), MSSA (22.6%), *E. faecalis* (19.4%), and *Streptococcus* spp. (9.7%) were the most isolated pathogens. Almost all patients (98.4%) received prior antibiotic treatment for a median of 9.5 days, followed in 60.5% of cases by a second regimen for a median of 24.5 days. DAL usually represented a sequential or consolidation therapy in hospitalised patients, with a single 1500 mg dose being the most frequent regimen. Surgery was undergone in 45.9% of cases, usually before DAL. The main reason for prescription was to accelerate the rate of discharge (95.2%), resulting in a median fourteen-day reduction in hospital stay. Overall clinical success in patients who completed the one-year follow-up was 95.9% [9].

An observational study enrolled 22 patients treated with DAL after previous antimicrobials, of whom three had IE. Overall, *S. aureus* and CoNS were the most isolated pathogens, and the success rate was 95% [110].

A single-centre retrospective experience described 10 IE cases (three native valve, five prosthetic, and two CIED IE) mainly caused by staphylococci and enterococci. A median of 2.5 DAL doses were administered after at least 2 weeks of antimicrobials. Microbiological cure was obtained in 70% of cases, but long-term mortality was high (60%) and two patients relapsed [111].

Another retrospective analysis included 102 individuals, 14 (13.7%) of them with IE. All received antibiotics before DAL for a median of 18.5 days. *S. aureus* was isolated in 70.6% of cases. IE patients had a DAL LD of 1500 mg followed by a range of one to six 1500 mg doses. Overall, 93.7% reached clinical and microbiological success, and hospitalization was reduced by a median of 14 days (range 7–84) [112].

Several other studies investigated DAL in poorly compliant people with IE including homeless people, people who inject drugs (PWID), and people with alcohol disorders. In the majority of cases, patients were treated with previous intravenous antimicrobial regimens and were unsuitable for OPAT. Overall, the clinical success of DAL use was high, ranging from 66% to 100% [113,114,115,116,117,118,119,120]. However, the number of patients lost at follow-up was not negligible.

Finally, several cases and case series have described prolonged DAL treatment in patients with IE, with conflicting results [121,122,123,124,125,126,127]. Among the seven individuals with IE included in the study of real-life experience by Bouza et al., DAL was mainly used as a targeted therapy and only one failure was recorded [128].

Some authors reviewed the clinical efficacy of DAL for IE, with an overall success rate ranging from 68% to 95% [129,130], but acknowledged that most of the evidence came from retrospective studies and that there was a huge heterogeneity in the population included (PWID, cardiac device-related IE), the definition of outcomes, the quality of studies, the indications, and the dosing strategies. Notably, only three cases of DAL resistance were detected [96]. Our search confirmed this landscape.

Overall, we analyzed 313 cases of IE treated with DAL (the most-used regimen was a 1500 mg single or repeated dose), caused mostly by *S. aureus* (with a slight predominance of MSSA), followed by CoNS. Native valves of the right side were predominantly involved but cases involving the left side, prosthetic valves, and CIEDs were reported as well. Previous antibiotic treatment before DAL was almost universal. Clinical and microbiological outcomes were generally positive although there was an elevated rate of patients lost to follow-up and the data are difficult to interpret because of high heterogeneity.

**Table 3:** Clinical studies investigating the treatment of infective endocarditis with dalbavancin.

Authors	Study Design	Endpoint	N° Patients/ IE Type	Pathogens	Dosage and Duration	Combination, Dosage	Outcomes	Safety
Bouza, E. et al., 2018 [128]	Multicentre retrospective study	Efficacy, tolerability, and cost reductions in people receiving DAL for various indications	69, mainly prosthetic joint infections (29%) and ABSSSI (21.7%) Previous therapy 97% 7 IE, type unspecified.	**IE subgroup:** CoNS (2), *Enterococcus* spp. (2), MRSA (1), *Streptococcus* spp. (1), negative culture (1)	Most common regimen: 1000 mg Day 1, then weekly 500 mg	Overall, 36.2%	Overall clinical success 84.1% and significant cost reduction **IE subgroup** Clinical success: 85.7%. Failure in 1 IE patient attributed to inadequate source control	Overall, AE in 13%. Most common AE: rash and tachycardia.
Tobudic, S. et al., 2018 [14]	Observational retrospective study DAL in IE mainly administered as OPAT	Clinical cure and safety	27 IE Previous therapy 88.9% 16 NVE, 6 PVE and 5 CIED-IE	*S. aureus* (33.3%), CoNS (22%), and *E. faecalis* (14.8%) main pathogens	Administered as twice-weekly regimen in 63.0% Median duration of 6 weeks (range, 1–30 weeks).	No	Clinical and microbiological success: 92.6%. Failure in 1 patient with MRSA CIED-IE and incomplete surgical control	2 AE: 1 nausea and vomiting after the second dose, therapy continued. 1 creatinine increase, resolved with dose reduction.
Bryson-Cahn, C. et al., 2019 [115]	Observational retrospective study on vulnerable patients *S. aureus* serious infection	Clinical response: any patient who had an FU visit within 1 year without evidence of ongoing/relapsed infection	32 infections (BSI 40.6%, osteoarticular 28%) Previous therapy 100%. 9 IE tricuspid NVE	2 IE MSSA 7 IE MRSA	22 received a single 1000 mg dose, 7 received 2 weekly doses	No	**IE subgroup:** Clinical response 5/9 Lost to FU 4/9	No AE reported
Bork, J.T. et al., 2019 [116]	Multicentre retrospective study on vulnerable patients Invasive Gram-positive infections	Clinical cure	45 infections (osteomyelitis 45%, endovascular 25%) Previous therapy 100%. 6 IE, type unspecified	MRSA (29%) and MSSA (21%) main pathogens	Median of 3 doses prescribed	6 patients with concomitant oral fluoroquinolone.	Overall, 30 day cure was achieved by 50% of patients with endovascular infection; >25% loss to FU. IE subgroup unspecified.	AEs documented in 6.7% (2 acute kidney injuries and 1 rash)
Dinh, A. et al., 2019 [108]	Multicentre retrospective study French national cohort	Clinical cure	75 infections (most frequent bone and joint 64%, endocarditis 25%). Previous therapy 98.7% 19 IE: 9 NVE and 10 PVE	*S. aureus* (51.4%) and CoNS (44.4%) main pathogens	In IE most frequent regimen was 1500 mg single or double dose	Overall, 45.3%, mainly rifampicin, cotrimoxazole, quinolones and tetracyclines	Overall, clinical cure 79%. **IE subgroup** Clinical cure: 72.2%	Five AE in the cohort (6.7%) with no treatment discontinuation
Hidalgo-Tenorio, C. et al., 2023 [9]	Multicentre retrospective study DAL as consolidation treatment	Effectiveness of DAL as consolidation therapy	124 IE (46.8% native valve, 43.6% prosthetic valve and 9.6% pacemaker lead IE). Previous therapy 100%.	CoNS (38.7%), MSSA (22.6%) *E. faecalis* (19.4%) and Streptococcus species (9.7%) the most isolated pathogens	Single 1500 mg dose the most prescribed DAL regimen (33.3%)	No data reported	Clinical success in subjects that completed the 1 year follow-up: 95.9% Mean reduction in hospital stay: 14 days.	AE in 3.2%
Morrisette, T. et al., 2019 [107]	Multicentre retrospective study DAL or ORI in various infections	Clinical success	56 infections (ABSSSI 36%, osteomyelitis 27%), 40 DAL, 14 ORI and 2 both. Previous therapy 91% 5 IE, type unspecified.	MSSA (25%), MRSA (19%) and *E. faecalis* (11%) main pathogens	No data reported	30% of the whole cohort (drugs unspecified)	**IE subgroup** Clinical success: 100% among the 3 evaluable IE	Mild AE in 11%.
Wunsch, S. et al., 2019 [109]	Multicentre retrospective study DAL as sequential treatment	Clinical success	101 infections (prosthetic joint 31%, osteomyelitis 30%, IE 25%) Previous therapy 100% 25 IE: 15 NVE, 6 PVE, 4 CIED-IE	CoNS (33%), MSSA (16%), MRSA (9%) main pathogens	In IE, 9 single 1500 mg dose and 1000 mg dose followed by 500 mg 1 week apart.	Overall, 64% of the cohort, mainly rifampicin (64%) and fluoroquinolones (15%)	Overall, clinical success 89%. **IE subgroup** Clinical success: 92%	Three AE in the cohort (3%), requiring treatment discontinuation
Ajaka, L. et al., 2020 [117]	Observational retrospective study in people with barriers to SoC	Cure: lack of clinical or microbiological persistent/recurrent infection within 90 days or negative BCs within 90 days after completion of DAL	28 infections (24 BSI and 4 IE) Previous therapy 100%. PWID 67% 4 IE, type unspecified	MRSA (39%) and MSSA (17%) main pathogens	LD of 1500 mg followed by 1 maintenance dose	No	Overall, 44% clinical cure, 33% failed treatment, and 22% lost to FU.	No data reported
Bai, F. et al., 2020 [106]	Multicentre retrospective study DAL in various infections	Clinical cure	206 infections (124 ABSSSI, 82 other site infection) Previous therapy 77.8% 6 IE, type unspecified.	MRSA (29%), CoNS (35%) and MSSA (17%) in the non-ABSSSI group.	Overall, single 1500 mg dose in 60.2%	In 37.2% of non-ABSSSI patients, mainly fluoroquinolones, rifampicin, and tetracycline	Overall clinical cure in non-ABSSSI 75%. **IE subgroup** Clinical cure: 83.3%	5.4% had an AE, mainly dermatologic. One serious AE (Stevens–Johnson).
Núñez-Núñez, M. et al., 2020 [110]	Observational prospective study. DAL as sequential treatment	Clinical success	22 infections (osteoarticular 46%, BSI 23%). Previous therapy 100%. 3 IE, type unspecified.	*S. aureus* (55%), CoNS (27%)	63% of the whole cohort received 1000 mg followed by 500 mg	No data reported	Overall, clinical success 95%	AE 1 (4.5%), infusion site reaction
Veve, M.P. et al., 2020 [119]	Observational retrospective study DAL vs. SOC	Incidence of infection-related readmission within 90 d of hospital discharge or outpatient DAL administration	215 infections (most common BSI, osteoarticular and IE) 70 DAL vs. 145 SoC Previous therapy 100%. IE 54: 9 DAL vs. 45 SOC	MRSA 82%	Most frequent regimen 2: 1500 mg doses 1 week apart	in 13% of DAL treated.	Overall, DAL was associated with lower 90-day infection-related readmissions and shorter length of stay.	AE 2.9% in the DAL group, 1 required discontinuation.
Durante-Mangoni, E. et al., 2021 [111]	Observational single-centre retrospective study DAL in IE	Clinical and microbiological cure	10 IE: 3 NVE, 5 PVE, 2 CIED-IE At least 2 weeks previous therapy 100%	Mainly caused by staphylococci and enterococci.	Median of 2.5 DAL doses per patient	No data reported	Clinical and microbiological cure 70%	1 AE (rash after the third dose) with treatment withdrawal
Arrieta-Loitegui, M. et al., 2022 [112]	Observational retrospective study DAL as sequential treatment	Clinical and microbiological cure	102 infections (SSTI 30%, BSI 15.7%, IE 13.7%) Previous therapy 100%. 14 IE, type unspecified	*S. aureus* in 70.6%	IE patients, 1500 mg as LD followed by a range of 1–6: 1500 mg doses	16.7%, mainly moxifloxacin and linezolid	Overall, clinical and microbiological success: 93.7%. Median reduction in hospitalization 14 days (range 7–84).	AE in 3.9%, 1 patient discontinued.
Taylor, K. et al., 2022 [114]	Observational retrospective study DAL as sequential treatment	Clinical success	48 infections (osteomyelitis 54%, IE 23%, BSI 15%). 11 IE, type unspecified. Previous therapy 100%	MRSA (42%) and MSSA (19%) main pathogens	Most patients received 1500 mg doses 44% 1 dose, 52% 2 doses.	27%, mainly rifampin and quinolones	Overall clinical success 85%. **IE subgroup:** Clinical success at 90 days 82%.	No AE reported
Lueking, R. et al., 2023 [120]	Observational retrospective study Vulnerable people receiving DAL	Clinical failure (not defined)	40 infections (BSI 67.5%, ABSSSI 45%) Previous therapy 100%. 4 IE, type unspecified	MRSA (57.5%) and MSSA (30%) main pathogens	Most frequent regimen 1500 mg single dose	In 15% of the whole cohort.	**IE subgroup:** Clinical success in all patients	AE in 5%
Vazquez Deida, A.A. et al., 2020 [118]	Case series Vulnerable people receiving DAL	N/A	27 infections (BSI 26%, IE 26%). Previous therapy 100% PWID 67% 9 right side IE	*S. aureus* 100% (48% MRSA).	Single DAL dose 7–10 days before the planned end of therapy	No	**IE subgroup:** Clinical success in 6/9 Estimated cost avoidance of USD 9600 per patient in the whole cohort	AE in 7.4% (mild events)
Guleri, A. et al., 2021 [113]	Case series DAL in IE	N/A	11 IE, 4 aortic NVE, 3 aortic PVE, 1 mitro-aortic NVE, 1 mitral NVE, 1 ICD-IE, 1 tricuspid NVE) Previous therapy 100%.	MSSA and *E. faecalis*, main pathogens	1 or 2: 1500 mg doses	9, mostly oral amoxicillin.	Clinical cure in all but one patient	No AE reported
Hitzenbichler, F. et al., 2021 [127]	Case series DAL after clearance of bacteraemia	N/A	4 IE 2 PVE 2 LVAD	MRSA *E. faecalis* *E. faecium*	Long-term suppressive DAL, various regimens	No	Clinical success with prolonged infection suppression in all IE cases	No AE reported
Steele, J.M. et al. 2018 [121]	Case report DNS strain	N/A	1 Tricuspid NVE	DNS MRSA	1000 mg LD, then 3 weekly 500 mg doses	No	Clinical and microbiological failure, bacteraemia relapse, isolation of a VISA and telavancin-non susceptible MRSA	No AE reported
Kussmann, M. et al., 2018 [125]	Case report	N/A	1 CIED-IE with incomplete PMK explantation	MRSA	Unspecified dosing 30 weekly administrations	No	Clinical and microbiological failure, bacteraemia relapse, isolation of a SCV strain teicoplanin-resistant and DAL non-susceptible	No AE reported
Howard-Anderson, J. et al., 2019 [122]	Case report Suppressive therapy	N/A	1 LVDA	MRSA	Weekly 1500 mg for 10 weeks, then 1500 mg biweekly. Total DAL exposure: 235 days	No	Clinical success with prolonged infection suppression	No AE reported
Spaziante, M. et al., 2019 [126]	Case report	N/A	1 Aortic PVE in a man with unacceptable perioperative risk	MRSE	1500 mg whenever serum bactericidal activity titers detected ≤ 1:8	No	Clinical and radiological improvement with no recurrence	No AE reported
Hakim, A. et al., 2020 [123]	Case report DAL as primary regimen	N/A	1 Tricuspid NVE	MSSA	1500 mg LD, followed by 5 weekly 500 mg doses	No	Clinical success	No AE reported
Teigell-Muñoz, F.J. et al., 2023 [124]	Case report DAL as consolidation therapy	N/A	1 Aortic NVE	*E. faecalis*	1000 mg single dose, after 4 weeks of therapy and valve replacement	No	Clinical success	No AE reported

Abbreviations: ABSSSI: acute bacterial skin and skin structure infection; AE: adverse event; BC: blood cultures; BSI, bloodstream infection; CIED: cardiovascular implantable electronic device; CoNS: coagulase-negative staphylococci; DAL: dalbavancin; IE: infectious endocarditis; IM: intramuscular; LD: loading dose; LVAD: left ventricular assist device; MRSA: methicillin-resistant S. aureus; MSSA: methicillin-susceptible S. aureus; N/A: not applicable; NVE: native valve endocarditis; OD: once daily; OPAT: outpatient parenteral antibiotic therapy; ORI: oritavancin; PVE: prosthetic valve endocarditis; PWID: people who inject drugs; SCV: small colony variant; SOC: standard of care. Definitions: Clinical cure/success was defined, unless otherwise specified, as resolution of clinical signs of infection; as absence of clinical signs of infection [107]; as no further evidence of infection or microbiological evidence of infection control (clearance of cultures) [106]; as improvement in lesions and resolution of signs and symptoms at end of treatment [105]; as completed treatment course without change or addition of antibiotic therapy, and with no additional antibiotics commenced within 48 h of discontinuation of the targeted antimicrobial therapy [109]; as no clinical, laboratory, or microbiological evidence of persistent or recurring infection during a 90 day follow-up [108]; as resolution of signs and symptoms of IE with negative BCs after end of therapy [110]; and as no need for additional therapy, and no additional positive cultures at 90 days [113]. Microbiological cure was defined as a documented negative blood culture result or BC clearance, unless otherwise specified.

### 3.4. Oritavancin

#### 3.4.1. Mechanism of Action and Indication

Oritavancin (ORI) is a second-generation semisynthetic lipoglycopeptide with an extensive tissue distribution, a high binding affinity for plasma proteins, and a long terminal half-life (393 h). With its concentration-dependent bactericidal action, it disrupts the membranes of Gram-positive bacteria causing depolarization and inhibits the production of cell wall peptidoglycan by binding either to D-Ala-D-Ala or to D-Ala-D-Lac residues [131]. This bactericidal action through multiple mechanisms is considered to confer a low probability of resistance development [130]. ORI acts against streptococci, as well as *S. aureus* and *S. epidermidis*, regardless of susceptibility to methicillin. Differently from DAL and telavancin, ORI retains activity against both VanA- and VanB-phenotype enterococci. In addition, it is active against VISA and vancomycin-resistant *S. aureus* (VRSA) [132].

ORI maintains activity inside the biofilms of MSSA, MRSA, and vancomycin-susceptible and resistant enterococci [133]. Notably, the activity of ORI is not limited to the extracellular environment but concentrates in lysosomes and effectively addresses pathogens persisting intracellularly, as occurs with the SCV phenotype [134].

The currently available evidence concerning ORI in vitro synergisms and experimental models of IE is discussed in Supplementary Material, Sections S4.1 and S4.2 [135,136,137,138,139].

In 2014 and 2015, ORI was approved by the FDA and EMA, respectively, for ABSSSI [140]. Similar to DAL, given its optimal spectrum, tissue penetration, prolonged half-life, and side effect profile, ORI was explored for multiple off-label indications in invasive Gram-positive infections [141].

#### 3.4.2. Clinical Evidence in Infective Endocarditis

Presently, data on ORI off-label use are limited, as shown in Table 4 [142].

In the multicentre retrospective cohort studied by Morrisette et al., 40 patients were treated with DAL, 14 were treated with ORI, and two were treated with both. In the whole cohort, five people had IE; however, unfortunately, it is not possible to distinguish how many received ORI. The success rate was 100% among the three IE cases analyzed [107].

A multicentre retrospective analysis was conducted among four hospitals and several clinics. Out of 75 patients receiving ORI, four had IE. The most common pathogens were MSSA and MRSA, and 13.3% of the population were PWID. In the whole cohort, the main reasons for ORI use were IV-line placement avoidance (61.3%) and social/insurance barriers (46.7%). Three patients with IE achieved clinical cure, the fourth was readmitted due to chest pain during the second infusion, subsequently attributed to cocaine use [11].

A retrospective single-centre analysis was performed on a very complex population (100% PWID, 70% with psychiatric illness, 67% homeless) treated with ORI. Two out of 23 patients had tricuspid IE. The first patient had MSSA and received 30 days of prior therapy followed by a single 1200 mg ORI dose and obtained clinical cure. The second had MRSA IE and, after 47 days of inpatient treatment, received two 1200 mg doses of ORI one week apart, but was finally recorded as a clinical failure [143]. Two single cases of IE treated with ORI reported clinical and microbiological success obtained after valve replacement surgery [144,145]. In a case series, after inpatient antibiotic therapy, five PWID with IE (two due to MSSA, two due to MRSA, one due to group A/F *Streptococcus*) were selected for ORI due to active illicit drug use and risk for IV-line manipulation. Clinical success was achieved by three patients, while two were lost to follow-up [146]

Overall, we retrieved only 13 IE cases of various types that were treated with ORI 1200 mg single or repeated doses, which were caused by staphylococci for the most part and frequently affected people with reduced compliance. Results were commonly good.

**Table 4:** Clinical studies investigating the treatment of infective endocarditis with oritavancin.

Authors	Study Design	Endpoint	N° Patients/ IE Type	Pathogens	Dosage and Duration	Combination, Dosage	Outcomes	Safety
Stewart, C.L. et al., 2017 [145]	Observational retrospective study ORI as an off-label indication	Clinical cure	10 infections (BSI 50%) 1 tricuspid NVE in a PWID with previous therapy: VAN (3 days), then CRO (4 days)	*Streptococcus agalactiae*	IE patient 1200 mg 1 dose and then discharged	No	Clinical failure with need for valve replacement 3 months after ORI administration	No AE reported
Ahiskali, A. et al., 2020 [143]	Observational retrospective study on a vulnerable population of PWID receiving ORI	Clinical cure	23 infections (BSI 50%) Previous therapy 100%. 2 IE, type unspecified	1 MSSA 1 MRSA	MSSA IE: single 1200 mg dose, MRSA IE: two 1200 mg doses	No	**IE subgroup:** Clinical cure 1 (MSSA), Clinical failure 1 (MRSA)	AE in 8.7%, mild
Brownell, L.E. et al., 2020 [11]	Multicentre observational retrospective study ORI as primary treatment	Clinical cure	75 infections (ABSSSI 49%) No previous treatment 4 IE, type unspecified	MSSA (31.5%) and MRSA (17.8%)	All patients included received initial 1200 mg dose followed by 1200 or 800 mg weekly	No data reported	**IE subgroup:** Clinical cure 75% Average hospital days avoided in IE: 18 d	AE in 12%, most commonly back pain with infusion. All resolved upon discontinuation
Salcedo, D.A.T. et al., 2018 [146]	Case series of Gram-positive IE in PWID	N/A	5 IE Previous therapy 100%.	MRSA (20%), MSSA (20%), *Streptococcus* (10%)	2 received 4 ORI doses, 3 received only 1 dose	No	Clinical cure: 3/5 Lost to FU: 2/5	AE in 1 patient (allergic reaction treated with oral prednisone)
Johnson, J.A. et al., 2015 [144]	Case report Limited treatment options	N/A	1 Aortic PVE	VR *E. faecium*.	1200 mg every other day for 3 doses, then weekly for 6 weeks, then 1200 mg biweekly for 10 weeks after recurrence and valve exchange	GEN for the first 4 days, discontinued due to renal toxicity	Recurrence after the first treatment course attributed to lack in source control. Clinical cure after valve exchange and a second prolonged course of ORI	Mild increase in transaminases

Abbreviations: ABSSSI: acute bacterial skin and skin structure infection; AE: adverse event; IE: infectious endocarditis; MRSA: methicillin-resistant *S. aureus*; MSSA: methicillin-susceptible *S. aureus*; N/A: not applicable; ORI: oritavancin; PVE: prosthetic valve endocarditis; NVE: native valve endocarditis; PWID: people who inject drugs; VR: vancomycin resistant; VAN: vancomycin; GEN: gentamycin; CRO: ceftriaxone, FU: follow-up. Definitions: Clinical cure was defined as the resolution of all clinical signs and symptoms of infection or without need for additional antimicrobial therapy following completion of ORI.

### 3.5. Old Antibiotics with a Renewed Interest: Fosfomycin

#### 3.5.1. Mechanism of Action and Indication

Fosfomycin (FOS) is a broad-spectrum bactericidal agent, with activity against several Gram-negative and Gram-positive pathogens, that enters the bacterial cell through the L-alpha-glycerophosphate and the hexose-6-phosphate transporter systems and acts by interfering with the formation of the peptidoglycan precursor uridine diphosphate N-acetylmuramic acid (UDP-MurNAc) [147]. This feature makes cross-resistance with other antibiotics highly uncommon [148].

Although discovered more than four decades ago, its use has only recently been repurposed for the treatment of severe infections caused by Gram-negative MDR [147,149,150,151] or Gram-positive pathogens such as MSSA/MRSA and VRE, showing promising results in terms of clinical efficacy and safety [10,148,152].

Indeed, its unique mechanism of action, along with its high level of in vitro synergism and its extensive tissue distribution, even in difficult-to-reach areas, renders FOS a very promising combination partner for the treatment of several infections, including IE [147,148].

Studies investigating FOS in vitro synergisms and experimental models of IE are shown in Supplementary Material, Sections S5.1 and S5.2 [153,154,155,156,157,158,159,160,161,162,163,164,165,166,167,168,169,170,171,172,173,174,175,176,177].

Current drug indications for FOS, namely infections for which no other antibiotics may be recommended, include complicated urinary tract infections, IE, bone and joint infections, pneumonia, skin and soft tissue infections, intra-abdominal infections, and meningitis, with or without bacteraemia [178].

#### 3.5.2. Clinical Evidence in Infective Endocarditis

Clinical experience concerning the possible role of FOS-containing combinations for the treatment of Gram-positive IE has accumulated over time. Translating from in vitro and in vivo experiments, the most studied combinations were DAP and FOS and imipenem and FOS (Table 5).

The first report concerning the combination of imipenem and FOS dates back to 1994 [179]. Subsequently, Del Rio et al. performed a clinical trial including adults receiving appropriate antibiotic therapy for MRSA bacteraemia or IE but who needed imipenem and FOS as rescue therapy because of persistent bacteraemia, unacceptable side effects of antibiotics, or relapse. Among the 16 patients included, 12 suffered from IE. Overall, the primary outcome (defined as negative blood cultures 72 h after the first dose) was reached in all the patients, with no breakthrough episodes of MRSA bacteraemia and an overall clinical success rate of 91.6% [180].

In 2018, Pericas et al. performed an RCT comparing patients receiving imipenem and FOS with VAN for the treatment of MRSA BSI, among whom eight had IE (four in each regimen). The primary endpoint was persistent bacteraemia at seven days while secondary endpoints were the clearance of blood cultures at 72 h after the initiation of study treatment, relapse of bacteraemia, and mortality. Persistent bacteraemia was absent and blood cultures at 72 h were negative in all patients receiving imipenem and FOS, while cure rates were similar between the two regimens (4/8 vs. 3/7 imipenem and FOS vs. VAN, respectively) [181].

Subsequently, Pujol and colleagues performed an RCT comparing DAP (10 mg/kg/24 h) and FOS (2 g every 6 h) with DAP alone (10 mg/kg/24 h) for the treatment of MRSA BSI. Of the 155 patients included, 112 underwent echocardiography and 18/112 (11.6%) had left-side IE. Combination therapy achieved treatment success in a higher number of patients, although it was not statistically significant (54.1% vs. 42%). Notably, microbiological failure was significantly lower in the combination arm than in the monotherapy arm (0% vs. 11.1%). After stratification for patients with or without IE, no differences were observed. On the other hand, side effects were higher in patients receiving DAP and FOS than those receiving DAP alone [10].

A post hoc analysis of the INSTINCT prospective cohort study, including 578 patients with *S. aureus* bacteraemia, among whome 129 had IE, evaluated combination therapy with either rifampin (*n* = 242) or FOS (*n* = 58) versus monotherapy. The authors found that combination therapy was associated with a better outcome than monotherapy, and this was also observed in the subgroup of patients with IE. No differences between the rifampin of FOS combinations were observed for 90 day mortality [182,183]. The DAP or VAN and FOS combination was also reported in the case reports and case series [184,185,186].

Overall, we analyzed 294 IE episodes, mostly caused by MRSA and treated mainly with FOS in combination with different ß-lactams or DAP/VAN. When the data were reported, the native or prosthetic valves of the left side were predominantly involved. Clinical and microbiological outcomes were generally positive, leading the DAP and FOS regimen to be included in the recent guidelines [5].

**Table 5:** Clinical studies investigating the treatment of infective endocarditis with fosfomycin.

Authors	Study Design	Endpoint	N° Patients/ IE Type	Pathogens	Dosage and Duration	Combination, Dosage	Outcomes	Safety
Del Rio, A. et al., 2014 [180]	Multicentre prospective clinical trial IMI + FOS as rescue therapy for MRSA BSI	Primary endpoints: negative BC at 72 h, clinical success ^§^ rate assessed at the test-of-cure visit in the ITT population	16 BSI 12 IE	MRSA	2 g/6 h * Median 28 d (SD 4–75)	IMI 1 g/6 h *	Overall, negative BC 72 h after the first dose in all the patients, No MRSAB breakthrough episodes, Clinical success: 91.6%, Mortality: 5 (31%), only 1 related to the infection or to the antibiotic therapy	5/16 (31%) 1: leukopenia 1: fungal BSI 3: sodium overload
Rieg, S. et al., 2017 [183]	Post hoc analysis of the INSTINCT prospective multicentre cohort study Patients with SAB	All-cause 30 d and 90 d mortality, death, or SAB-related late complications within 180 days	964 BSI (452 monotherapy and 512 combination) FOS was used in 99/512 (19%) 121 (12.6%) IE [20/512 (4.4%) monotherapy, 101/452 (19.7%) combination]	MRSA 108/964 (11.2%) MSSA 856/964 (88.8%)	5 g/8 h Median duration 14 d (IQR 7–26, range 1–66)	MSSA: FLU, VAN, TEC, DAP MRSA: VAN, TEIC, DAP, LNZ	Overall, 30 d mortality: monotherapy 82/443 (18.5%), combination 93/509 (18.3%), (*p* = 1) 90 d mortality: monotherapy 140/436 (32.1%), combination 156/503 (31%), (*p* = 0.87) SAB-related late complications within 180 d: monotherapy 25/428 (5.8%), combination 19/490 (3.9%), (*p* = 0.18) No specific outcomes in patients receiving FOS	No data reported
Pericas, J.M. et al., 2018 [181]	Open-label randomised clinical trial IMI + FOS vs. VAN for MRSA BSI	Primary endpoint: persistent bacteraemia at 7 d Secondary endpoints: negative BC at 72 h after the initiation of study treatment, relapse of BSI, mortality	15 BSI 8 IE FOS + IMI (*n* = 8) (4 complicated BSI, 4 IE: 2 NVE, 2 PVE) VAN (*n* = 7) (3 complicated BSI, 1 NVE, 3 CIED-IE)	MRSA	2 g/6 h **EI group**, VAN: mean 35.7 d (range 27–42), IMI + FOS: mean 18.2 d (range 4–51) Complicated bacteraemia VAN: mean 18.3 d (range 17–21), IMI + FOS: mean 27.2 d (range 15–42)	IMI 1 g/6 h VAN 30–45 mg/kg/24 h (divided into 2–3 doses, trough levels ≥ 15 mg/L)	Overall, all patients in the FOS + IMI arm had negative BC at 3 days Cure rates: IMI + FOS 4 (50%) VAN 3 (43%) In-hospital mortality: IMI + FOS 3 (37.5%), VAN 1 (14.2%) Persistent bacteriemia: IMI + FOS 0, VAN 1 (14.2%) Relapse: IMI + FOS 0, VAN 1 (14.2%)	IMI + FOS: 1 salt overload VAN: 1 renal toxicity
Rieg, S. et al., 2020 [182]	Post hoc analysis of the INSTINCT prospective multicentre cohort study Patients with SAB	All-cause 90 d mortality, death, or SAB-related late complications within 180 days	578 BSI [313 combination with RIF (*n* = 242) or FOS (*n* = 58) and 265 monotherapy 129 IE, 23% NVE, 7,1% of CIED or vascular grafts or PVE	MSSA 250 (94%) monotherapy 264 (84%) combination MRSA 15 (6%) monotherapy 49 (16%) combination	5 g/8 h Median 23 d (IQR 13–33)	MSSA: FLU or DAP MRSA: VAN, TEIC, DAP, LNZ	Overall, all-cause 90 d mortality: 190/565 (34%), Death or SAB-related late complications within 180 d: 45% [52% (132/255) monotherapy vs. 39% (115/297) combination], Combination therapy was associated with a better outcome than monotherapy (HR 0.65, 95% CI 0.46–0.92), especially in implanted foreign devices. **IE subgroup:** 90 d mortality: 16/32 (50%) monotherapy, 27/81 (33%) RIF, 4/11 (36%) FOS	No data reported
Pujol, M. et al., 2021 [10]	Randomised clinical trial DAP + FOS vs. DAP for MRSA BSI	Treatment success 6 weeks after the end of therapy	155 BSI 18 left-side IE	MRSA	2 g/6 h DAP + FOS: median 14 d (IQR 11–21)	DAP 10 mg/kg/24 h DAP Median 14 days (IQR 10–18.5)	Overall, treatment success °: DAP + FOS 40/74 (54.1%), DAP 34/81 (42.0%) (*p* = 0.135) Microbiological failure °°: DAP + FOS 0, DAP 9/81 (11.1%) (*p* = 0.003) Persistent bacteraemia at 7 d: DAP + FOS 0, DAP 5/81 (6.2%) Complicated bacteraemia: DAP + FOS 12/74 (16.2%), DAP 26/81 (32.1%) (*p* = 0.022) No differences were observed in patients **with or without IE**	DAP + FOS 13/74 (17.6%) DAP 4/81 (4.9%) (*p* = 0.018)
Aoyagi, S. et al., 1994 [179]	Case report	N/A	1 IE on ventricular patch graft	MRSA	300 mg/6 h (paediatric dosage) 24 d	IMI 125 mg/6 h (paediatric dosage)	Clearance of bacteraemia: 24 h from FOS start Symptom-free during 12 months of follow-up	No data reported
Chen, L.Y. et al., 2011 [184]	Case report	N/A	1 CIED-IE plus osteomyelitis	DNS MRSA	6 g/6 h 56 d	DAP 9 mg/kg/24 h, followed by 12 mg/kg/24 h	Clearance of bacteraemia: 7 d Symptom free during 12 months of follow-up	No AE reported
Mirò, J.M. et al., 2012 [185]	Case series Failure with high-dose DAP or VAN	N/A	3 IE (1 aortic PVE, 2 left-sided NVE)	1 MSSA (PVE) 2 MRSA (NVE)	2 g/6 h 6 weeks	DAP 10 mg/kg/24 h	Clearance of bacteraemia Alive at 6 months (*n* = 1) and 12 months (*n* = 2) FU No need of surgery	No AE reported
Vergara-Lopez, S. et al., 2015 [186]	Case report	N/A	1 Aortic NVE	MRSE + carbapenem-resistant *Klebsiella* *oxytoca*	4 g/6 h 28 d	VAN (1 g/12 h) AMK (1 g/24 h)	Clearance of bacteraemia Complete disappearance of the vegetation at echocardiography	Self-limited hypokalaemia

Abbreviations: CIED-EI: cardiovascular implantable electronic device endocarditis; IE: infective endocarditis; FOS: fosfomycin; DAP: daptomycin; MRSA: methicillin-resistant *S. aureus*; MRSE: methicillin-resistant *S. epidermidis*; VAN: vancomycin; AMK: amikacin; IMI: imipenem; BC: blood culture; ITT: intention-to-treat; BSI: bloodstream infection; INSTINCT: invasive stapyhlococcus aureus infection; CohorT; SAB: *S. aureus* bacteraemia; MSSA: methicillin-susceptible *S. aureus*; FLU: flucloxacillin; TEC: teicoplanin; LNZ: linezolid; PVE: prosthetic valve endocarditis; MRSAB: methicillin-resistant *S. aureus* bacteraemia. Definitions: Clinical success was defined as clinical improvement with resolution of all signs and symptoms of infection during treatment or at the end of therapy unless otherwise specified. Notes: ^§^: Treatment was classified as clinically successful when the patient was alive, lacked signs or symptoms of infection, and had sterile blood cultures at the test-of-cure visit. Failure was defined as death, positive blood cultures, or discontinuation of FOS plus IMI because of persistent bacteraemia or AEs; *: Between 2001 and 2005, all patients received VAN as initial therapy; this was continued, and FOS and IMI were added. After 2006, FOS and IMI were administered instead of the initial antibiotic regimen, which included either DAP at 6–10 mg/kg or VAN; °: Treatment success was considered when patient was alive and had resolution of clinical manifestations of infection and negative blood cultures at test-of-cure after completion of therapy; °°: Microbiological failure was considered in the case of persistent bacteraemia, recurrent bacteraemia, and the emergence of resistance to study drugs during treatment.

## 4. Oral Strategies

There has been great interest in oral step-down strategies for the treatment of IE; however, most of the evidence comes from old trials or retrospective and observational studies, with controversial results [187,188,189,190,191].

It is only with the recent multicentre unblinded non-inferiority POET trial that the long-lasting paradigm of treating IE always (and only) with prolonged intravenous treatment has changed. Indeed, this trial was able to show that, in stable patients with *Streptococcus* spp., *E. faecalis*, *S. aureus*, or CoNS left-side IE, changing to oral antibiotics after an initial phase of at least 10 days of intravenous treatment was not inferior to continued intravenous antibiotic treatment [192]. However, it should be noted that only 22% of the enrolled patients had *S. aureus* IE, only a small percentage of patients with IV drug use was included, and, although it was not an exclusion criterion, no patients with MRSA-IE or other antibiotic-resistant phenotypes were enrolled, rendering the results not fully generalizable. Among the several proposed schemes, the most commonly used during the trial were dicloxacillin or amoxicillin and rifampicin for *S. aureus*, linezolid and rifampicin or fusidic acid for CoNS, amoxicillin and linezolid or moxifloxacin for *E. faecalis*, and amoxicillin and rifampicin or moxifloxacin for streptococci [192].

The five-year follow-up of the same trial demonstrated that the composite primary outcome (defined as death from any cause, unplanned cardiac surgery, embolic events, and relapse of a blood culture result positive for the primary pathogen) occurred in 32.8% and 45.2% of step-down and continued intravenous treatment groups, respectively. Interestingly, this difference was mainly driven by a lower incidence of death from any cause in the first group, while no differences were observed for the other parameters of the composite outcome [193].

Taken together, these findings appear somehow reassuring concerning the potential role of oral step-down therapy for the treatment of selected and stable patients with left-side IE.

A recent published multicentre retrospective cohort confirmed this potential role, with no significant difference between the IV-only and oral groups in terms of clinical success at 90 days. Moreover, the oral group patients had significantly fewer adverse events. In this cohort, the most commonly used therapy was 600 mg of oral linezolid twice a day with or without rifampin [13]. Focused on *E. faecalis* IE, a small case series proposed an interesting oral step-down combination therapy with amoxicillin/clavulanate and cefditoren [194]. In a study published in 2009, the authors proposed an early switch from intravenous VAN to oral linezolid for the treatment of MRSA IE only after an aggressive surgical approach. This oral step-down showed a reduction in recurrences, hospitalization, and economic costs [195].

Possible oral strategies for the sequential step-down therapy are shown in Table 6.

Additional results will be available after the completion of the RODEO trials, which will compare oral switch and intravenous antibiotic therapies in patients with staphylococcal and streptococcal/enterococcal left-sided IE (RODEO-1 and RODEO-2, respectively) [196].

Tedizolid phosphate (TDZ) is a second-generation form of oxazolidinone. Compared to linezolid, TDZ is administered once daily with less myelotoxicity and fewer drug–drug interactions. There is no clinical data on TDZ in human IE. Based on in vitro and in vivo activity, TDZ may be considered a possible agent for the treatment of IE only as a sequential therapy after IV treatment with other agents in patients not eligible for other regimens [197,198]. Due to the lack of clinical evidence, no recommendation on its use for IE may be given and it remains a potential candidate without sufficient clinical evidence.

**Table 6:** Possible oral strategies for sequential step-down therapy. The decision to use sequential step-down oral therapy must only be made if the patient is clinically stable, and the choice of drug regimen must always be based on the antimicrobial susceptibility of the bacteria isolated (adapted from [192]).

Bacteria	Oral Antibiotic Strategies for Step-Down Treatment ^#^			
MSSA/ MS-CONS	Dicloxacillin + rifampicin/fusidic acid	Levofloxacin/moxifloxacin + rifampicin/fusidic acid	Linezolid monotherapy or linezolid + adjunctive therapy	TMP-SMX + adjunctive therapy
MRSA	Linezolid *°			
MR CONS	Linezolid + levofloxacin/moxifloxacin	Levofloxacin/moxifloxacin + rifampicin/fusidic acid/clindamycin	Linezolid monotherapy or linezolid + rifampicin	TMP-SMX + adjunctive therapy
Oral Streptococci/ *Streptococcus* spp.	Amoxicillin monotherapy or amoxicillin + rifampicin	Moxifloxacin + rifampicin/clindamycin/amoxicillin	Linezolid monotherapy or linezolid + rifampicin/clindamycin/amoxicillin	Moxifloxacin + linezolid
*E. faecalis*	Amoxicillin/clavulanate + cefditoren ° or amoxicillin + rifampicin	Moxifloxacin + Amoxicillin/rifampicin	Linezolid monotherapy or linezolid + amoxicillin/rifampicin	Moxifloxacin + linezolid
GISA (hVISA, VISA, DNS)	**NOT RECOMMENDED** (No data available)			
*E. faecium*				
VVR *Enterococcus* spp.				

Legend: ^#^ Only used in stable patients and always based on the antimicrobial susceptibility; * after surgical intervention; ° need of future investigations; adjunctive therapy: rifampicin, clindamycin, or fusidic acid. MSSA: methicillin-susceptible *S. aureus*; MRSA: methicillin-resistant *S. aureus*; CoNS: coagulase-negative *Staphylococci*; VISA: vancomycin-intermediate *S. aureus*; hVISA: heterogeneus vancomycin-intermediate *S. aureus*; DNS: Damptomycin-unsusceptible, VR: vancomycin-resistant; MS: methicillin-susceptible; MR: methicillin-resistant.

## 5. New Therapeutic Strategies: Considerations for Their Optimal Use in IE

IE is a major public health challenge associated with high morbidity and mortality [2]. Recently released guidelines have introduced several updates regarding its prevention, diagnosis, and management [5]. From a therapeutic point of view, by introducing the possibility of a step-down oral strategy in selected stable patients, the new recommendations divided the antibiotic treatment of IE into two phases: the first one (critical phase), which can last up to 2 weeks, includes in-hospital intravenous therapy using combinations of rapidly bactericidal antibiotics to destroy planktonic bacteria; after this period, selected clinically stable patients can end the antibiotic treatment at home with intravenous (OPAT) or oral antibiotic regimens for up to 6 weeks (continuation phase) [5].

Compared to the previous 2015 guidelines, the choice of antibiotics in the first phase has been expanded with the introduction of new molecules and combinations, including, among others, the combination DAP and FOS or CPT for MSSA and MRSA. As for the consolidation phase, weekly DAL schemes as an alternative to oral or OPAT strategies have been considered [5,6].

In the present manuscript, we reviewed the currently available in vitro, in vivo, and clinical evidence on the use of new beta-lactams (CPT, BPR), long-acting agents (DAL and ORI), and the repurposed drug FOS for their possible use in the treatment of IE.

As shown in Figure 1A, the evidence supporting the use of CPT and BPR (alone or in combination with DAP), FOS, and long-acting DAL and ORI for staphylococcal IE has accumulated over time [7,9,10,11,14,39,65,68,182]. Despite exhibiting pre-clinical evidence, the new beta-lactams and their associations with DAP have garnered less clinical evidence for MSSA IE, which has been limited to case series/case reports (shown as yellow or yellow/green colour, Figure 1A); this could be possibly explained by the strong efficacy of the currently recommended agents (i.e., cefazolin) [39,65].

In contrast, the combination of DAP and FOS has gained clinical evidence supporting its use thanks to the RCT by Pujol et al. (shown as green colour, Figure 1A). Likewise, for MRSA the combinations of DAP and FOS and DAP and CPT gained pre-clinical and clinical evidence supported by the RCTs by Pujol et al. and by Geriak et al., respectively, as well as by observational studies [8,10]. Choosing one of these two regimens over the other should be based on several factors, including beta-lactam allergies, which favuor DAP and FOS, or the risk of exacerbating cardiac or renal failure with the sodium overload associated with FOS, a condition favouring DAP and CPT.

According to the promising results of the recent ERADICATE RCT, which included 20 patients with *S. aureus* IE, a green/yellow colour was attributed to BPR for *S. aureus*, similar to the evidence available for BPR and DAP (Figure 1A) [39]. However, we believe that the use of BPR for the treatment of staphylococcal IE (alone or in combination with DAP) will increase over time.

As for the long-acting agents, so far, the majority of clinical evidence is available for DAL, especially with regard to MSSA and MRSA (shown as green colour, Figure 1A). Nevertheless, the most effective administration schedule is still not clear, since high variability is present in the literature concerning the number of dosages, their interval, and the duration of therapy [96]. Consensus agreement in this setting is highly warranted. In contrast, ORI’s clinical evidence for MSSA and MRSA is limited only to case reports/case series (shown as green/yellow colour, Figure 1A), probably due to its only recent introduction in the market [142]. However, based on ORI in vitro activity towards these pathogens, it is likely that additional clinical evidence will accumulate in the coming years, positioning ORI as a potential additional therapeutic strategy in the treatment of IE.

Although supported by less clinical evidence than *S. aureus*, the same considerations mentioned above may be drawn for CoNS (Figure 1A).

Since strong and consolidated clinical evidence exists concerning the management of beta-lactam-susceptible *E. faecalis* and streptococcal IE, we only reviewed the available literature data on the potential use of new agents for IE.

As shown by Figure 1B, most of the evidence regarding CPT+/−DAP or the long-acting drugs for streptococcal IE comes from evidence supported by in vitro activity, animal studies, and case reports/series (shown as yellow/green colour, Figure 1B), while, for BPR or beta-lactams and FOS, evidence is supported by in vitro activity and animal studies in the absence of clinical evidence for their effectiveness against streptococcal IE (shown as yellow colour, Figure 1B). As for *E. faecalis* IE, beta-lactams and FOS or CPT+/−DAP present poor in vitro data and no in vivo and clinical evidence and therefore are shown as yellow/red colour (Figure 1B).

Likewise, the combinations FOS or BPR and DAP for streptococcal IE present an absence of in vitro, animal, and clinical data (shown as red colour, Figure 1B). BPR in combination with ampicillin was investigated in a small series of *E. faecalis* IE cases, showing promising results [40] (shown as yellow/green colour, Figure 1B).

Much less knowledge has been gained concerning *E. faecium* or VAN-R enterococcal IE, where the currently available evidence only comes from in vitro and animal studies, while clinical evidence is still lacking (yellow/red or red colour, Figure 1B). In this regard, a recent study showed that the combination of high-dose daptomycin with FOS improved the survival rate of patients with VRE-BSI compared to daptomycin alone. However, only one case of IE was included, which was treated with DAP alone [152]. Additional clinical evidence on the potential role of DAP and FOS in the setting of IE is therefore needed.

The only regimen whose evidence is supported also by clinical evidence is DAL for *E. faecalis* IE, which therefore may be considered as a possible strategy after the initial phase of in-hospital intravenous therapy when other options are not feasible and may be associated with cost-effectiveness and reductions in hospitalization lengths [9,110]. Although active in vitro, ORI suffers from a lack or paucity (only case reports/case series) of clinical evidence concerning *E. faecium* and *E. faecalis* IE. However, similar to what we have hypothesised concerning staphylococcal IE, we believe that, as evidence accumulates, ORI will be an important therapeutic step-down regimen for enterococcal IE.

## 6. Conclusions

In conclusion, while for streptococcal, MSSA, and *E. faecalis* IE the use of new drugs/strategies may be only limited to particular cases since the currently recommended regimens are highly effective and well tolerated, the treatment of staphylococcal IE cases, in particular those sustained by MRSA and methicillin-resistant CoNS, may benefit from new strategies including: (i) CPT/BPR, alone or in combination with DAP, (ii) FOS in association with DAP, or (iii) long-acting DAL and ORI as step-down treatments.

Overall, only poor evidence is currently available concerning the potential roles of these new strategies for the treatment of *E. faecium* IE (only limited to cases when current recommended regimens are not feasible or effective) and vancomycin-resistant enterococcal IE, which represents one of the most difficult to treat conditions. We strongly believe that additional studies aiming to fill this gap are warranted.

A multidisciplinary approach to IE is highly recommended in order to use, as best as possible, the new therapeutic weapons we have at our disposal, which should be defended in accordance with antimicrobial stewardship principles.

## Data Availability

The data presented in this review are retrieved and summarised from the different published studies available.

## Supplementary Materials

The following supporting information can be downloaded at: https://www.mdpi.com/article/10.3390/jcm12247693/s1, supplementary sections on studies investigating in vitro synergisms of new antimicrobials and experimental animal models of IE.
