# Supplementary material for: New Antimicrobials and New Therapy Strategies for Endocarditis: Weapons That Should Be Defended
Source: J Clin Med. 2023 Dec 14;12(24):7693. doi: 10.3390/jcm12247693 (PMC10743892; doi:10.3390/jcm12247693)
Supplement: Supplementary file 1 [file jcm-12-07693-s001.zip › jcm-2674419-supplementary.pdf]

Supplementary Material

Studies Investigating In Vitro Synergisms of New Antimicrobials and Experimental Animal Models of IE

1. Ceftobiprole

1.1 In Vitro Synergism

BPR synergism with different antimicrobials such as aminoglycosides, plazomicin, levofloxacin, and rifampin has been evaluated in several studies [1–15]. Campanile et al. specifically studied the in vitro synergism of BPR with daptomycin, linezolid, levofloxacin, rifampin, or piperacillin/tazobactam against different 46 Gram-positive pathogens, such as *S. aureus*, CoNS, and *Enterococcus spp.*, and found that BPR exhibited synergism with daptomycin against all Gram-positive isolates and only against *Enterococcus spp*. with levoxacin [7]; additionally, treatment with linezolid was only effective against four isolates belonging to different species. These results were consistent with the previous study by Barber et al., who demonstrated that the combination of BPR and daptomycin (DAP) was the most potent in vitro antibiotic combination against methicillin-resistant *S. aureus* (MRSA) strains with varying degrees of vancomycin (VAN) susceptibility [14]. Interestingly, BPR maintains in vitro activity against DAP- and BPR-resistant MRSA clinical isolates and even enhanced DAP binding to the cell membrane in DAP-resistant strains by altering the charge and membrane fluidity [8].

BPR also showed activity against ampicillin-sensitive *E. faecalis* [1]; however, resistance to ampicillin in *E. faecalis* and *E. faecium* correlated with reduced susceptibility and resistance to BPR, respectively [3]. The data provided by Deshpande et al, who evaluated the combination of BPR and gentamicin against *Enterococcus spp.*, is also of interest [3]. The authors showed how BPR and gentamicin exhibited synergism against 62.5% of VAN-susceptible or intermediate *E. faecium*, while this combination was indifferent in the only two (37,5%) *E. faecalis* samples tested. Arias et al. (2007) also observed synergy between BPR and aminoglycosides (streptomycin) against two strains of beta lactamase-producing *E. faecalis* and synergy between BPR and gentamicin against a strain of VAN-resistant *E. faecalis* (vanB), but not against the VanA sample they tested [15].

Aware of evidence regarding the synergistic effect of beta-lactam antibiotics and DAP against VAN-resistant *E. faecium* and *E. faecalis* (VRE), Werth et al. found that BPR and DAP were synergistic against four out of six strains of VAN-resistant *E. faecium* and *E. faecalis* with varying susceptibilities to DAP [16]. Additionally, exposure to BPR reduced the minimum inhibitory concentration (MIC) of DAP in all tested strains.

Peiffer-Smadja et al. investigated the in vitro bactericidal activity of amoxicillin combined with various cephalosporins including BPR against 10 *E. faecalis* strains isolated from patients with IE caused by the same pathogen. The authors found that the combinations of amoxicillin and BPR and ceftaroline (CPT) and amoxicillin consistently exhibited lower bactericidal activity compared to combinations with other cephalosporins such as cefazolin, cefotaxime, ceftriaxone, and cefepime [9] .

1.2 Animal Studies

BPR was first investigated by Enteza JM et al. in an experimental rat model of MRSA IE, showing that BPR was able to sterilize >90% of cardiac vegetation [10].

Subsequently, BPR was evaluated in a rabbit model of aortic valve IE due to methicillin-resistant and vancomycin-intermediate *S. aureus* (VISA). While BPR was as effective as VAN against the MRSA strains examined in the study, it was more effective than VAN against VISA strains [12]. Likewise, Tattevin et al. demonstrated the potency of BPR and its superiority to VAN, DAP, and linezolid in an animal model of IE caused by MRSA, showing that there were significantly fewer residual organisms in vegetation in BPR-treated rabbits than in any other treatment groups [11].

2. Ceftaroline

2.1 In Vitro Synergism

The association between CPT and VAN or DAP shows in vitro synergism compared with any agent alone against MRSA or daptomycin-unsusceptible (DNS) MRSA [17,18], including VISA isolates [19]. Werth et al. found that the association between DAP and CPT resulted in significantly improved killing compared to DAP or VAN alone against DNS, VISA, and MRSA strains. Although DAP reached bactericidal activity faster than CPT alone, the association of DAP and CPT was significantly faster than DAP alone against all strains [20]. While most beta-lactams, when combined with VAN, showed synergism against vancomycin-sensitive MRSA (VSSA), heterogenous VISA (hVISA), and VISA strains, VAN/CPT had the most beneficial effect in terms of its time-killing curve [21]. Eliazar et al. found that the combination DAP/CPT was more active than VAN/CPT or any agent alone against DNS MRSA and VISA bacteriemia

isolates [22]. Considering DAP-resistant *S. aureus*, CFT showed synergy with DAP or VAN, which was most evident in cases of lower CPT MICs [23].

Several in vitro studies showed a superior synergic effect of CPT combined with ampicillin (AMP) against *E. faecalis* isolates if compared with AMP alone or AMP/gentamicin [24,25]. Likewise, CPT/AMP was more active against *E. faecalis* than AMP/ceftriaxone (CRO) [24]. Cusumano et al. described an in vitro synergism of ertapenem (ERT) or meropenem (MEM) in combination with CPT against *E. faecalis* isolates: the combination of MEM/CPT in vitro was as active as AMP and CRO [26]. Interestingly, CPT reduced the MICs of DAP in VRE strains in vitro, suggesting its potential use in vivo [27].

The most widely accepted hypothesis regarding the synergism between AMP and cephalosporins is complementary penicillin-binding protein (PBP) saturation [28]. Indeed, CPT binds PBP 2 and 3 with an increased affinity for PBP2a, while AMP binds PBP 4 and 5 [29]. Moreover, CPT increases cell membrane fluidity and decreases the cell surface charge. Interestingly, the mechanism of synergism between DAP and CPT could restore DAP sensibility in DNS *E. faecium* strains. Thus, DAP binding on cell surfaces is significantly increased by CPT in DNS *E. faecium* strains [30]. The enhanced DAP binding on cell surfaces due to CPT was also found for MRSA [18].

2.2 Animal Studies

The bactericidal activity of CPT has been largely demonstrated by Jaqueline and colleagues in several in vivo studies using rabbit IE models. The authors reported that in MSSA, MRSA, hGISA, and MR-GISA rabbit IE models, CPT alone showed a higher four-day bactericidal activity than all glycopeptides, DAP, and linezolid, with a higher volume of sterile vegetation in all rabbits treated with CPT [31–33]. Furthermore, CPT showed more potent activity than VAN or linezolid against vancomycin-sensitive or -resistant *E. faecalis* IE [34]. Finally, a recent study on a murine model showed that the CPT efficacy against MRSA BSI is not inferior after 24 h of treatment with VAN and DAP. The CPT effectiveness was also demonstrated in hVISA and DNS strains, showing its potential use against difficult to treat *S. aureus* bacteraemia [35].

3. Dalbavancin

3.1 In Vitro Synergism

Synergy was analyzed for dalbavancin (DAL) and nine different antimicrobials against MRSA, MSSA, VISA, methicillin-resistant S. epidermidis (MRSE), vancomycin-susceptible *E. faecalis*, *S. pneumoniae*, and *S. pyogenes*. The results showed no antagonism, but full synergism was present only with oxacillin (the β-lactam representative). Gentamycin and VAN had frequent partial synergy (60%), followed by clindamycin (40%), DAP, levofloxacin and quinopristin/dalfopristin (30% each), and rifampicin (10%). Linezolid offered the least active combination (80% indifferent) [36]. More recently, DAL combination with β-lactams was evaluated against different phenotypes of *S. aureus*: MRSA, VISA, hVISA, DAP non-susceptible (DNS), and linezolid-resistant. All agents reduced DAL MICs, but the time-kill curves showed that DAL with cefazolin, cefepime, and ertapenem had synergy against all isolates; DAL with ceftaroline had synergy against all strains but one MRSA strain; DAL with oxacillin was not synergic against two MRSA strains and one hVISA strain. The authors suggested that agents with PBP-1 affinity may be more effective for DAL synergism [37].

3.2 Animal Studies

The applicability of DAL in IE was first investigated by Candiani et al. in a rat staphylococcal IE model where, at lower dosages, it was shown to be as effective as teicoplanin and VAN in bacterial load reduction [38]. Subsequently, DAL was tested in an in vivo rabbit model of IE caused by glycopeptide-sensitive and glycopeptide-intermediate *S. aureus* (GISA) isolated from a human before and after exposure to VAN and teicoplanin, respectively. The activity was not influenced by the reduced glycopeptide susceptibility and no DAL-resistant strain was detected [39].

4. Oritavancin

4.1 In Vitro Synergism

ORI was combined with other agents against one vancomycin-susceptible *E. faecium* strain, and four VRE strains. Synergy was shown in one VRE strain treated with ORI and gentamycin, while antagonism emerged for ORI with DAP

(two VRE) and ORI with rifampicin (one VRE) [40]. Recently, the combination of ORI with fosfomycin (FOS) demonstrated synergism in eight out of ten VRE (five VanA and five VanB) isolates and showed an addictive effect in the remaining two. The combination restored FOS-susceptibility in 85% of FOS-resistant isolates [41].

4.2 Animal Studies

In 1998, ORI was tested on a rabbit model of MRSA left-sided IE. Compared to VAN, after a four-day treatment course, there were no significant differences in cleared bacteremia, reduced vegetation, or tissue bacterial counts [42]. In rabbit models of IE, ORI was also compared to VAN and teicoplanin against three different strains of *E. faecalis* (glycopeptide-susceptible, VanA and VanB). Its bactericidal activity was not decreased by the glycopeptide resistance, regardless of the phenotype [43]. In a similar experiment, the in vivo combination with gentamicin was synergistic and bactericidal against the three *E. faecalis* strains and prevented the emergence of mutants [44].

5. Old Antibiotic With a Renewed Interest: Fosfomycin

5.1 In Vitro Synergism

A recent systematic review comprising a total of 1087 Gram-positive isolates investigated the rate of synergism of DAP-containing regimens using in vitro and in vivo studies. A total of 14 studies evaluating the DAP and FOS combination were evaluated. The authors found that the combination was synergistic against most *S. aureus* isolates (55.4%). In particular, the combination was synergistic against all the MSSA isolates [45]. Likewise, DAP and FOS was bactericidal against the majority of MRSA strains at both standard and high bacterial inocula (106 and 108 CFU/mL, respectively) [46]. Less recently, Debbia et al. showed a synergism rate of 80-90% of FOS and DAP against a collection of 50 staphylococcal strains, including MRSE [47]. This synergistic activity may be explained by the PBP-1 inhibition exerted by FOS and by fosfomycin's ability to modify cell wall protein compositions [46,48–50]. The combination of DAP and FOS has also been shown to be synergistically active against a DAP-resistant strain in an in vitro and in vivo model of MRSA IE [49].

Five in vitro studies evaluated DAP and FOS against 22 isolates of *Enterococcus spp.* (fourteen *E. faecalis*, eight *E. faecium*): the most frequent interaction was synergism (63.6%), which was slightly higher for VAN-resistant than for susceptible strains. Fosfomycin and DAP also exhibited a greater efficacy against *E. faecalis* biofilm formation than the single drugs alone [45,51].

Conflicting results concerning the effect of FOS in combination with cephalosporins against clinical isolates of *Staphylococcus spp.* have been reported, with synergism rates ranging from 16% to 100% [51]; in particular, a study reported that results were different between FOS and ceftriaxone against *S. epidermidis* if checkerboard or killing studies were used (16% and 83.3%, respectively) [52]. However, a large number of available reports of in vitro and in vivo studies showed synergistic activity of FOS and different beta-lactams against MRSA, in particular with regard to imipenem–FOS, which was found to be the most active combination against MRSA and one GISA strain [53–57]. Likewise, meropenem and imipenem showed a synergism of 90–100% against *S. epidermidis* [55,57]. The mechanism of synergy between FOS and beta-lactams is not well understood. However, molecular studies showed that FOS and imipenem significantly decreased PBP1, PBP2 (but not PBP2a), and PBP3 synthesis, thus increasing the effect of imipenem on MRSA and GISA strains [55].

The combination of ceftriaxone and FOS showed a high in vitro synergistic effect against a collection of *E. faecalis* strains isolated from patients with IE, with a synergy rate of 55.6% [58]. Descorouez et al. (2013) showed full synergism of amoxicillin and FOS against four strains of VRE, while antagonistic effects were found against six biofilm-producer *E. faecalis* isolates with the combination of FOS and ampicillin [59,60].

As for *Streptococcus spp.*, less data is described; however, Gonzalez Moreno et al., in an in vitro biofilm model with microcalorimetry, showed a delay of 8 h in the production of heat if FOS was added to benzylpenicillin against *S. anginosus*, *S. agalactiae*, or *S. oralis* compared with untreated controls, while less recent studies showed a synergistic rate of 23% and 32% in in vitro experiments with FOS combined with penicillin G and ampicillin, respectively [58,59,61–63].

5.2 Animal Studies

Experimental animal models of IE with FOS have been described for many years, showing encouraging results [46,47,62,64]. Likewise, FOS's anti-biofilm activity has been shown in several models of infections [65–67].

In an experimental model of rabbit MRSA IE, DAP and FOS significantly improved the efficacy of DAP monotherapy at 6 mg/kg/day in terms of both the proportion of sterile vegetation and a decrease in the density of bacteria within the vegetation. Interestingly, DAP and FOS was as effective as DAP monotherapy at 10 mg/kg/day [46]. The same authors in a rabbit model of MSSA IE showed that adding FOS or cloxacillin to DAP significantly improved the efficacy of DAP

in sterilizing vegetation and showed better activity than cloxacillin alone. No recovered isolates showed increased DAP MIC [47].

Rice et al. evaluated the combination of DAP and FOS against a strain of *E. faecalis* with high-level gentamicin resistance in a rat IE model; the authors found an absence of superiority of the combination therapy over the monotherapy. No antagonism was found when considering *Enterococcus spp.* models [63].

Del Rio et al. showed that the combination of imipenem and FOS was highly synergistic against MRSA in an experimental model of IE, with a percentage of sterile vegetation of 73% in the combination group compared to 0% and 7% in cases using FOS or imipenem alone, respectively [56]. Notably, the combined regimen avoided the development of resistance to FOS, which was, in contrast, observed in 42% of isolates recovered from the FOS monotherapy arm. Vicente et al. (1981) showed a 100% rate of synergistic interaction between FOS and cefoxitim in an experimental model of IE due to *S. sanguis*, with a significant reduction in log10 CFU per gram of vegetation in the combination group compared to each drug alone [62].

References

1. Pfaller, M.A.; Flamm, R.K.; Mendes, R.E.; Streit, J.M.; Smart, J.I.; Hamed, K.A.; Duncan, L.R.; Sader, H.S. Ceftobiprole Activity against Gram-Positive and -Negative Pathogens Collected from the United States in 2006 and 2016. *Antimicrob. Agents Chemother.* **2019**, *63*, e01566-18. https://doi.org/10.1128/AAC.01566-18
2. López Díaz, M.C.; Ríos, E.; Rodríguez-Avial, I.; Simaluiza, R.J.; Picazo, J.J.; Culebras, E. In-Vitro Activity of Several Antimicrobial Agents against Methicillin-Resistant Staphylococcus Aureus (MRSA) Isolates Expressing Aminoglycoside-Modifying Enzymes: Potency of Plazomicin Alone and in Combination with Other Agents. *Int. J. Antimicrob. Agents* **2017**, *50*, 191–196. https://doi.org/10.1016/j.ijantimicag.2017.01.039.
3. Deshpande, L.M.; Jones, R.N. Bactericidal Activity and Synergy Studies of BAL9141, a Novel Pyrrolidinone-3-Ylidenemethyl Cephem, Tested against Streptococci, Enterococci and Methicillin-Resistant Staphylococci. *Clin. Microbiol. Infect. Off. Publ. Eur. Soc. Clin. Microbiol. Infect. Dis.* **2003**, *9*, 1120–1124. https://doi.org/10.1046/j.1469-0691.2003.00765.x.
4. Rouse, M.S.; Steckelberg, J.M.; Patel, R. In Vitro Activity of Ceftobiprole, Daptomycin, Linezolid, and Vancomycin against Methicillin-Resistant Staphylococci Associated with Endocarditis and Bone and Joint Infection. *Diagn. Microbiol. Infect. Dis.* **2007**, *58*, 363–365. https://doi.org/10.1016/j.diagmicrobio.2007.02.010.
5. Leonard, S.N.; Cheung, C.M.; Rybak, M.J. Activities of Ceftobiprole, Linezolid, Vancomycin, and Daptomycin against Community-Associated and Hospital-Associated Methicillin-Resistant Staphylococcus Aureus. *Antimicrob. Agents Chemother.* **2008**, *52*, 2974–2976. https://doi.org/10.1128/AAC.00257-08.
6. Rodríguez-García, R.; Rodríguez-Esteban, M.Á.; García-Carús, E.; Telenti, M.; Fernández, J. In Vitro Activity of Ceftaroline and Ceftobiprole against Clinical Isolates of Gram-Positive Bacteria from Infective Endocarditis: Are These Drugs Potential Options for the Initial Management of This Disease? *Diagn. Microbiol. Infect. Dis.* **2020**, *98*, 115153. https://doi.org/10.1016/j.diagmicrobio.2020.115153.
7. Campanile, F.; Bongiorno, D.; Mongelli, G.; Zanghì, G.; Stefani, S. Bactericidal Activity of Ceftobiprole Combined with Different Antibiotics against Selected Gram-Positive Isolates. *Diagn. Microbiol. Infect. Dis.* **2019**, *93*, 77–81. https://doi.org/10.1016/j.diagmicrobio.2018.07.015.
8. Bongiorno, D.; Mongelli, G.; Stefani, S.; Campanile, F. Genotypic Analysis of Italian MRSA Strains Exhibiting Low-Level Ceftaroline and Ceftobiprole Resistance. *Diagn. Microbiol. Infect. Dis.* **2019**, *95*, 114852. https://doi.org/10.1016/j.diagmicrobio.2019.06.004.
9. Peiffer-Smadja, N.; Guillotel, E.; Luque-Paz, D.; Maataoui, N.; Lescure, F.-X.; Cattoir, V. In Vitro Bactericidal Activity of Amoxicillin Combined with Different Cephalosporins against Endocarditis-Associated Enterococcus Faecalis Clinical Isolates. *J. Antimicrob. Chemother.* **2019**, *74*, 3511–3514. https://doi.org/10.1093/jac/dkz388.
10. Entenza, J.M.; Veloso, T.R.; Vouillamoz, J.; Giddey, M.; Majcherczyk, P.; Moreillon, P. In Vivo Synergism of Ceftobiprole and Vancomycin against Experimental Endocarditis Due to Vancomycin-Intermediate Staphylococcus Aureus. *Antimicrob. Agents Chemother.* **2011**, *55*, 3977–3984. https://doi.org/10.1128/AAC.00402-11.
11. Tattevin, P.; Basuino, L.; Bauer, D.; Diep, B.A.; Chambers, H.F. Ceftobiprole Is Superior to Vancomycin, Daptomycin, and Linezolid for Treatment of Experimental Endocarditis in Rabbits Caused by Methicillin-Resistant Staphylococcus Aureus. *Antimicrob. Agents Chemother.* **2010**, *54*, 610–613. https://doi.org/10.1128/AAC.00886-09.
12. Chambers, H.F. Evaluation of Ceftobiprole in a Rabbit Model of Aortic Valve Endocarditis Due to Methicillin-Resistant and Vancomycin-Intermediate Staphylococcus Aureus. *Antimicrob. Agents Chemother.* **2005**, *49*, 884–888. https://doi.org/10.1128/AAC.49.3.884-888.2005.
13. Lin, G.; Ednie, L.M.; Appelbaum, P.C. Antistaphylococcal Activity of ACHN-490 Tested Alone and in Combination with Other Agents by Time-Kill Assay. *Antimicrob. Agents Chemother.* **2010**, *54*, 2258–2261. https://doi.org/10.1128/AAC.01350-09.
14. Barber, K.E.; Werth, B.J.; Ireland, C.E.; Stone, N.E.; Nonejuie, P.; Sakoulas, G.; Pogliano, J.; Rybak, M.J. Potent Synergy of Ceftobiprole plus Daptomycin against Multiple Strains of Staphylococcus Aureus with Various Resistance Phenotypes. *J. Antimicrob. Chemother.* **2014**, *69*, 3006–3010. https://doi.org/10.1093/jac/dku236.
15. Arias, C.A.; Singh, K.V.; Panesso, D.; Murray, B.E. Time-Kill and Synergism Studies of Ceftobiprole against Enterococcus Faecalis, Including Beta-Lactamase-Producing and Vancomycin-Resistant Isolates. *Antimicrob. Agents Chemother.* **2007**, *51*, 2043–2047. https://doi.org/10.1128/AAC.00131-07.

16. Werth, B.J.; Barber, K.E.; Ireland, C.E.; Rybak, M.J. Evaluation of Ceftaroline, Vancomycin, Daptomycin, or Ceftaroline plus Daptomycin against Daptomycin-Nonsusceptible Methicillin-Resistant Staphylococcus Aureus in an in Vitro Pharmacokinetic/Pharmacodynamic Model of Simulated Endocardial Vegetations. *Antimicrob. Agents Chemother.* **2014**, *58*, 3177–3181. https://doi.org/10.1128/AAC.00088-14.
17. Kaushik, D.; Rathi, S.; Jain, A. Ceftaroline: A Comprehensive Update. *Int. J. Antimicrob. Agents* **2011**, *37*, 389–395. https://doi.org/10.1016/j.ijantimicag.2011.01.017.
18. Werth, B.J.; Vidaillac, C.; Murray, K.P.; Newton, K.L.; Sakoulas, G.; Nonejuie, P.; Pogliano, J.; Rybak, M.J. Novel Combinations of Vancomycin plus Ceftaroline or Oxacillin against Methicillin-Resistant Vancomycin-Intermediate Staphylococcus Aureus (VISA) and Heterogeneous VISA. *Antimicrob. Agents Chemother.* **2013**, *57*, 2376–2379. https://doi.org/10.1128/AAC.02354-12.
19. Tran, N.; Rybak, M.J. β-Lactam Combinations with Vancomycin Show Synergistic Activity against Vancomycin-Susceptible Staphylococcus Aureus, Vancomycin-Intermediate S. Aureus (VISA), and Heterogeneous VISA. *Antimicrob. Agents Chemother.* **2018**, *62*, e00157-18. https://doi.org/10.1128/AAC.00157-18.
20. Dhand, A.; Bayer, A.S.; Pogliano, J.; Yang, S.-J.; Bolaris, M.; Nizet, V.; Wang, G.; Sakoulas, G. Use of Antistaphylococcal β-Lactams to Increase Daptomycin Activity in Eradicating Persistent Bacteremia Due to Methicillin-Resistant Staphylococcus Aureus: Role of Enhanced Daptomycin Binding. *Clin. Infect. Dis. Off. Publ. Infect. Dis. Soc. Am.* **2011**, *53*, 158–163. https://doi.org/10.1093/cid/cir340.
21. Eliazar, J.; Johnson, T.; Chbib, C. Pre-Clinical Impact of the Synergistic Mechanism of Daptomycin and Ceftaroline on Patients with Methicillin-Resistant Staphylococcus Aureus Bacteremia Infections. *Curr. Rev. Clin. Exp. Pharmacol.* **2021**, *16*, 296–299. https://doi.org/10.2174/1574884715666210108103813.
22. Hutton, M.A.; Sundaram, A.; Perri, M.B.; Zervos, M.J.; Herc, E.S. Assessment of Invitrosynergy of Daptomycin or Vancomycin plus Ceftaroline for Daptomycin Non-Susceptible Staphylococcus Aureus. *Diagn. Microbiol. Infect. Dis.* **2020**, *98*, 115126. https://doi.org/10.1016/j.diagmicrobio.2020.115126.
23. Werth, B.J.; Shireman, L.M. Pharmacodynamics of Ceftaroline plus Ampicillin against Enterococcus Faecalis in an In Vitro Pharmacokinetic/Pharmacodynamic Model of Simulated Endocardial Vegetations. *Antimicrob. Agents Chemother.* **2017**, *61*, e02235-16. https://doi.org/10.1128/AAC.02235-16.
24. Thieme, L.; Klinger-Strobel, M.; Hartung, A.; Stein, C.; Makarewicz, O.; Pletz, M.W. In Vitro Synergism and Anti-Biofilm Activity of Ampicillin, Gentamicin, Ceftaroline and Ceftriaxone against Enterococcus Faecalis. *J. Antimicrob. Chemother.* **2018**, *73*, 1553–1561. https://doi.org/10.1093/jac/dky051.
25. Cusumano, J.A.; Daffinee, K.E.; Piehl, E.C.; García-Solache, M.; Desbonnet, C.; Rice, L.B.; LaPlante, K.L. Meropenem plus Ceftaroline Is Active against Enterococcus Faecalis in an In Vitro Pharmacodynamic Model Using Humanized Dosing Simulations. *Antimicrob. Agents Chemother.* **2022**, *66*, e0042622. https://doi.org/10.1128/aac.00426-22.
26. Smith, J.R.; Barber, K.E.; Raut, A.; Aboutaleb, M.; Sakoulas, G.; Rybak, M.J. β-Lactam Combinations with Daptomycin Provide Synergy against Vancomycin-Resistant Enterococcus Faecalis and Enterococcus Faecium. *J. Antimicrob. Chemother.* **2015**, *70*, 1738–1743. https://doi.org/10.1093/jac/dkv007.
27. Mainardi, J.L.; Gutmann, L.; Acar, J.F.; Goldstein, F.W. Synergistic Effect of Amoxicillin and Cefotaxime against Enterococcus Faecalis. *Antimicrob. Agents Chemother.* **1995**, *39*, 1984–1987. https://doi.org/10.1128/AAC.39.9.1984.
28. EMA Zinforo. Available online: https://www.ema.europa.eu/en/medicines/human/EPAR/zinforo (accessed on 11 September 2023).
29. Sakoulas, G.; Moise, P.A.; Casapao, A.M.; Nonejuie, P.; Olson, J.; Okumura, C.Y.M.; Rybak, M.J.; Kullar, R.; Dhand, A.; Rose, W.E.; et al. Antimicrobial Salvage Therapy for Persistent Staphylococcal Bacteremia Using Daptomycin plus Ceftaroline. *Clin. Ther.* **2014**, *36*, 1317–1333. https://doi.org/10.1016/j.clinthera.2014.05.061.
30. Jacqueline, C.; Amador, G.; Batard, E.; Le Mabecque, V.; Miègeville, A.-F.; Biek, D.; Caillon, J.; Potel, G. Comparison of Ceftaroline Fosamil, Daptomycin and Tigecycline in an Experimental Rabbit Endocarditis Model Caused by Methicillin-Susceptible, Methicillin-Resistant and Glycopeptide-Intermediate Staphylococcus Aureus. *J. Antimicrob. Chemother.* **2011**, *66*, 863–866. https://doi.org/10.1093/jac/dkr019.
31. Jacqueline, C.; Caillon, J.; Batard, E.; Le Mabecque, V.; Amador, G.; Ge, Y.; Biek, D.; Potel, G. Evaluation of the in Vivo Efficacy of Intramuscularly Administered Ceftaroline Fosamil, a Novel Cephalosporin, against a Methicillin-Resistant Staphylococcus Aureus Strain in a Rabbit Endocarditis Model. *J. Antimicrob. Chemother.* **2010**, *65*, 2264–2265. https://doi.org/10.1093/jac/dkq328.
32. Jacqueline, C.; Caillon, J.; Le Mabecque, V.; Miègeville, A.-F.; Ge, Y.; Biek, D.; Batard, E.; Potel, G. In Vivo Activity of a Novel Anti-Methicillin-Resistant Staphylococcus Aureus Cephalosporin, Ceftaroline, against Vancomycin-Susceptible and -Resistant Enterococcus Faecalis Strains in a Rabbit Endocarditis Model: A Comparative Study with Linezolid and Vancomycin. *Antimicrob. Agents Chemother.* **2009**, *53*, 5300–5302. https://doi.org/10.1128/AAC.00984-09.
33. García, P.; Moscoso, M.; Fernández, M.C.; Fuentes-Valverde, V.; Pérez, A.; Bou, G. Comparison of the in Vivo Efficacy of Ceftaroline Fosamil, Vancomycin and Daptomycin in a Murine Model of Methicillin-Resistant Staphylococcus Aureus Bacteraemia. *Int. J. Antimicrob. Agents* **2023**, *62*, 106836. https://doi.org/10.1016/j.ijantimicag.2023.106836.
34. Jacqueline, C.; Caillon, J.; Le Mabecque, V.; Miègeville, A.-F.; Hamel, A.; Bugnon, D.; Ge, J.Y.; Potel, G. In Vivo Efficacy of Ceftaroline (PPI-0903), a New Broad-Spectrum Cephalosporin, Compared with Linezolid and Vancomycin against Methicillin-Resistant and Vancomycin-Intermediate Staphylococcus Aureus in a Rabbit Endocarditis Model. *Antimicrob. Agents Chemother.* **2007**, *51*, 3397–3400. https://doi.org/10.1128/AAC.01242-06.
35. Luther, M.K.; Rice, L.B.; LaPlante, K.L. Ampicillin in Combination with Ceftaroline, Cefepime, or Ceftriaxone Demonstrates Equivalent Activities in a High-Inoculum Enterococcus Faecalis Infection Model. *Antimicrob. Agents Chemother.* **2016**, *60*, 3178–3182. https://doi.org/10.1128/AAC.03126-15.

36. Johnson, D.M.; Fritsche, T.R.; Sader, H.S.; Jones, R.N. Evaluation of Dalbavancin in Combination with Nine Antimicrobial Agents to Detect Enhanced or Antagonistic Interactions. *Int. J. Antimicrob. Agents* **2006**, *27*, 557–560. https://doi.org/10.1016/j.ijantimicag.2005.12.015.
37. Xhemali, X.; Smith, J.R.; Kebriaei, R.; Rice, S.A.; Stamper, K.C.; Compton, M.; Singh, N.B.; Jahanbakhsh, S.; Rybak, M.J. Evaluation of Dalbavancin Alone and in Combination with β-Lactam Antibiotics against Resistant Phenotypes of Staphylococcus Aureus. *J. Antimicrob. Chemother.* **2019**, *74*, 82–86. https://doi.org/10.1093/jac/dky376.
38. Candiani, G.; Abbondi, M.; Borgonovi, M.; Romanò, G.; Parenti, F. In-Vitro and in-Vivo Antibacterial Activity of BI 397, a New Semi-Synthetic Glycopeptide Antibiotic. *J. Antimicrob. Chemother.* **1999**, *44*, 179–192. https://doi.org/10.1093/jac/44.2.179.
39. Lefort, A.; Pavie, J.; Garry, L.; Chau, F.; Fantin, B. Activities of Dalbavancin In Vitro and in a Rabbit Model of Experimental Endocarditis Due to Staphylococcus Aureus with or without Reduced Susceptibility to Vancomycin and Teicoplanin. *Antimicrob. Agents Chemother.* **2004**, *48*, 1061–1064. https://doi.org/10.1128/AAC.48.3.1061-1064.2004.
40. Wu, T.; Meyer, K.; Harrington, A.T.; Danziger, L.H.; Wenzler, E. In Vitro Activity of Oritavancin Alone or in Combination against Vancomycin-Susceptible and -Resistant Enterococci. *J. Antimicrob. Chemother.* **2019**, *74*, 1300–1305. https://doi.org/10.1093/jac/dkz010.
41. Lagatolla, C.; Mehat, J.W.; La Ragione, R.M.; Luzzati, R.; Di Bella, S. In Vitro and In Vivo Studies of Oritavancin and Fosfomycin Synergism against Vancomycin-Resistant Enterococcus Faecium. *Antibiot. Basel Switz.* **2022**, *11*, 1334. https://doi.org/10.3390/antibiotics11101334.
42. Kaatz, G.W.; Seo, S.M.; Aeschlimann, J.R.; Houlihan, H.H.; Mercier, R.C.; Rybak, M.J. Efficacy of LY333328 against Experimental Methicillin-Resistant Staphylococcus Aureus Endocarditis. *Antimicrob. Agents Chemother.* **1998**, *42*, 981–983. https://doi.org/10.1128/AAC.42.4.981.
43. Saleh-Mghir, A.; Lefort, A.; Petegnief, Y.; Dautrey, S.; Vallois, J.M.; Le Guludec, D.; Carbon, C.; Fantin, B. Activity and Diffusion of LY333328 in Experimental Endocarditis Due to Vancomycin-Resistant Enterococcus Faecalis. *Antimicrob. Agents Chemother.* **1999**, *43*, 115–120. https://doi.org/10.1128/AAC.43.1.115.
44. Lefort, A.; Saleh-Mghir, A.; Garry, L.; Carbon, C.; Fantin, B. Activity of LY333328 Combined with Gentamicin in Vitro and in Rabbit Experimental Endocarditis Due to Vancomycin-Susceptible or -Resistant Enterococcus Faecalis. *Antimicrob. Agents Chemother.* **2000**, *44*, 3017–3021. https://doi.org/10.1128/AAC.44.11.3017-3021.2000.
45. Antonello, R.M.; Principe, L.; Maraolo, A.E.; Viaggi, V.; Pol, R.; Fabbiani, M.; Montagnani, F.; Lovecchio, A.; Luzzati, R.; Di Bella, S. Fosfomycin as Partner Drug for Systemic Infection Management. A Systematic Review of Its Synergistic Properties from In Vitro and In Vivo Studies. *Antibiot. Basel Switz.* **2020**, *9*, 500. https://doi.org/10.3390/antibiotics9080500.
46. García-de-la-Mària, C.; Gasch, O.; García-Gonzalez, J.; Soy, D.; Shaw, E.; Ambrosioni, J.; Almela, M.; Pericàs, J.M.; Tellez, A.; Falces, C.; et al. The Combination of Daptomycin and Fosfomycin Has Synergistic, Potent, and Rapid Bactericidal Activity against Methicillin-Resistant Staphylococcus Aureus in a Rabbit Model of Experimental Endocarditis. *Antimicrob. Agents Chemother.* **2018**, *62*, e02633-17. https://doi.org/10.1128/AAC.02633-17.
47. García-de-la-Mària, C.; Gasch, O.; Castañeda, X.; García-González, J.; Soy, D.; Cañas, M.-A.; Ambrosioni, J.; Almela, M.; Pericàs, J.M.; Téllez, A.; et al. Cloxacillin or Fosfomycin plus Daptomycin Combinations Are More Active than Cloxacillin Monotherapy or Combined with Gentamicin against MSSA in a Rabbit Model of Experimental Endocarditis. *J. Antimicrob. Chemother.* **2020**, *75*, 3586–3592. https://doi.org/10.1093/jac/dkaa354.
48. Berti, A.D.; Sakoulas, G.; Nizet, V.; Tewhey, R.; Rose, W.E. β-Lactam Antibiotics Targeting PBP1 Selectively Enhance Daptomycin Activity against Methicillin-Resistant Staphylococcus Aureus. *Antimicrob. Agents Chemother.* **2013**, *57*, 5005–5012. https://doi.org/10.1128/AAC.00594-13.
49. Berti, A.D.; Theisen, E.; Sauer, J.-D.; Nonejuie, P.; Olson, J.; Pogliano, J.; Sakoulas, G.; Nizet, V.; Proctor, R.A.; Rose, W.E. Penicillin Binding Protein 1 Is Important in the Compensatory Response of Staphylococcus Aureus to Daptomycin-Induced Membrane Damage and Is a Potential Target for β-Lactam-Daptomycin Synergy. *Antimicrob. Agents Chemother.* **2016**, *60*, 451–458. https://doi.org/10.1128/AAC.02071-15.
50. Gasch, O.; Pillai, S.K.; Dakos, J.; Miyakis, S.; Moellering, R.C.; Eliopoulos, G.M. Daptomycin In Vitro Activity against Methicillin-Resistant Staphylococcus Aureus Is Enhanced by d-Cycloserine in a Mechanism Associated with a Decrease in Cell Surface Charge. *Antimicrob. Agents Chemother.* **2013**, *57*, 4537–4539. https://doi.org/10.1128/AAC.00799-13.
51. Antonello, R.M.; Canetti, D.; Riccardi, N. Daptomycin Synergistic Properties from in Vitro and in Vivo Studies: A Systematic Review. *J. Antimicrob. Chemother.* **2022**, *78*, 52–77. https://doi.org/10.1093/jac/dkac346.
52. Courcol, R.J.; Martin, G.R. In-Vitro Activity of the Combination of Ceftriaxone and Fosfomycin against Staphylococci. *J. Antimicrob. Chemother.* **1987**, *19*, 276–278. https://doi.org/10.1093/jac/19.2.276.
53. Duez, J.M.; Kohli, E.; Pechinot, A.; Tremeaux, J.C.; Kazmierczak, A. [Combination between fosfomycin and oxacillin or cefotaxime against methicillin-resistant Staphylococci and Enterococci]. *Pathol. Biol.* **1983**, *31*, 515–518.
54. Utsui, Y.; Ohya, S.; Magaribuchi, T.; Tajima, M.; Yokota, T. Antibacterial Activity of Cefmetazole Alone and in Combination with Fosfomycin against Methicillin- and Cephem-Resistant Staphylococcus Aureus. *Antimicrob. Agents Chemother.* **1986**, *30*, 917–922. https://doi.org/10.1128/AAC.30.6.917.
55. Grif, K.; Dierich, M.P.; Pfaller, K.; Miglioli, P.A.; Allerberger, F. In Vitro Activity of Fosfomycin in Combination with Various Antistaphylococcal Substances. *J. Antimicrob. Chemother.* **2001**, *48*, 209–217. https://doi.org/10.1093/jac/48.2.209.
56. del Río, A.; García-de-la-Mària, C.; Entenza, J.M.; Gasch, O.; Armero, Y.; Soy, D.; Mestres, C.A.; Pericás, J.M.; Falces, C.; Ninot, S.; et al. Fosfomycin plus β-Lactams as Synergistic Bactericidal Combinations for Experimental Endocarditis Due to Methicillin-Resistant and Glycopeptide-Intermediate Staphylococcus Aureus. *Antimicrob. Agents Chemother.* **2016**, *60*, 478–486. https://doi.org/10.1128/AAC.02139-15.

57. Guggenbichler, J.P.; Berchtold, D.; Allerberger, F.; Bonatti, H.; Hager, J.; Pfaller, W.; Dierich, M.P. In Vitro and in Vivo Effect of Antibiotics on Catheters Colonized by Staphylococci. *Eur. J. Clin. Microbiol. Infect. Dis. Off. Publ. Eur. Soc. Clin. Microbiol.* **1992**, *11*, 408–415. https://doi.org/10.1007/BF01961855.
58. Farina, C.; Russello, G.; Chinello, P.; Pasticci, M.B.; Raglio, A.; Ravasio, V.; Rizzi, M.; Scarparo, C.; Vailati, F.; Suter, F.; et al. In Vitro Activity Effects of Twelve Antibiotics Alone and in Association against Twenty-Seven Enterococcus Faecalis Strains Isolated from Italian Patients with Infective Endocarditis: High in Vitro Synergistic Effect of the Association Ceftriaxone-Fosfomycin. *Chemotherapy* **2011**, *57*, 426–433. https://doi.org/10.1159/000330458.
59. Tang, H.-J.; Chen, C.-C.; Zhang, C.-C.; Su, B.-A.; Li, C.-M.; Weng, T.-C.; Chiang, S.-R.; Ko, W.-C.; Chuang, Y.-C. In Vitro Efficacy of Fosfomycin-Based Combinations against Clinical Vancomycin-Resistant Enterococcus Isolates. *Diagn. Microbiol. Infect. Dis.* **2013**, *77*, 254–257. https://doi.org/10.1016/j.diagmicrobio.2013.07.012.
60. Descourouez, J.L.; Jorgenson, M.R.; Wergin, J.E.; Rose, W.E. Fosfomycin Synergy in Vitro with Amoxicillin, Daptomycin, and Linezolid against Vancomycin-Resistant Enterococcus Faecium from Renal Transplant Patients with Infected Urinary Stents. *Antimicrob. Agents Chemother.* **2013**, *57*, 1518–1520. https://doi.org/10.1128/AAC.02099-12.
61. Gonzalez Moreno, M.; Trampuz, A.; Di Luca, M. Synergistic Antibiotic Activity against Planktonic and Biofilm-Embedded Streptococcus Agalactiae, Streptococcus Pyogenes and Streptococcus Oralis. *J. Antimicrob. Chemother.* **2017**, *72*, 3085–3092. https://doi.org/10.1093/jac/dkx265.
62. Vicente, M.V.; Olay, T.; Rodríguez, A. Experimental Endocarditis Caused by Streptococcus Sanguis: Single and Combined Antibiotic Therapy. *Antimicrob. Agents Chemother.* **1981**, *20*, 10–14. https://doi.org/10.1128/AAC.20.1.10.
63. Olay, T.; Rodríguez, A.; Oliver, L.E.; Vicente, M.V.; Quecedo, M.C. Interaction of Fosfomycin with Other Antimicrobial Agents: In Vitro and in Vivo Studies. *J. Antimicrob. Chemother.* **1978**, *4*, 569–576. https://doi.org/10.1093/jac/4.6.569.
64. Rice, L.B.; Eliopoulos, C.T.; Yao, J.D.; Eliopoulos, G.M.; Moellering, R.C. In Vivo Activity of the Combination of Daptomycin and Fosfomycin Compared with Daptomycin Alone against a Strain of Enterococcus Faecalis with High-Level Gentamicin Resistance in the Rat Endocarditis Model. *Diagn. Microbiol. Infect. Dis.* **1992**, *15*, 173–176. https://doi.org/10.1016/0732-8893(92)90045-u.
65. Garrigós, C.; Murillo, O.; Lora-Tamayo, J.; Verdaguer, R.; Tubau, F.; Cabellos, C.; Cabo, J.; Ariza, J. Fosfomycin-Daptomycin and Other Fosfomycin Combinations as Alternative Therapies in Experimental Foreign-Body Infection by Methicillin-Resistant Staphylococcus Aureus. *Antimicrob. Agents Chemother.* **2013**, *57*, 606–610. https://doi.org/10.1128/AAC.01570-12.
66. Mihailescu, R.; Furustrand Tafin, U.; Corvec, S.; Oliva, A.; Betrisey, B.; Borens, O.; Trampuz, A. High Activity of Fosfomycin and Rifampin against Methicillin-Resistant Staphylococcus Aureus Biofilm in Vitro and in an Experimental Foreign-Body Infection Model. *Antimicrob. Agents Chemother.* **2014**, *58*, 2547–2553. https://doi.org/10.1128/AAC.02420-12.
67. Oliva, A.; Furustrand Tafin, U.; Maiolo, E.M.; Jeddari, S.; Bétrisey, B.; Trampuz, A. Activities of Fosfomycin and Rifampin on Planktonic and Adherent Enterococcus Faecalis Strains in an Experimental Foreign-Body Infection Model. *Antimicrob. Agents Chemother.* **2014**, *58*, 1284–1293. https://doi.org/10.1128/AAC.02583-12.
